# Autonomous Exploration of Small Bodies Toward Greater Autonomy for Deep Space Missions

**DOI:** 10.3389/frobt.2021.650885

**Published:** 2021-11-01

**Authors:** Issa A. D. Nesnas, Benjamin J. Hockman, Saptarshi Bandopadhyay, Benjamin J. Morrell, Daniel P. Lubey, Jacopo Villa, David S. Bayard, Alan Osmundson, Benjamin Jarvis, Michele Bersani, Shyam Bhaskaran

**Affiliations:** ^1^ Mobility and Robotics Systems Section, Jet Propulsion Laboratory, California Institute of Technology, Pasadena, CA, United States; ^2^ Mission Design and Navigation Section, Jet Propulsion Laboratory, California Institute of Technology, Pasadena, CA, United States; ^3^ Ann and H.J. Smead Aerospace Engineering Sciences Department, University of Colorado Boulder, Boulder, CO, United States; ^4^ Guidance and Control Section, Jet Propulsion Laboratory, California Institute of Technology, Pasadena, CA, United States; ^5^ University of Southern California, Los Angeles, CA, United States; ^6^ The University of Sydney, Camperdown, NSW, Australia; ^7^ Sant’Anna School of Advanced Studies, Pisa, Italy

**Keywords:** spacecraft autonomy, autonomous small-body navigation, autonomous approach and landing, small-body autonomy, small-sat autonomy, pole and shape estimation, small-body feature tracking

## Abstract

Autonomy is becoming increasingly important for the robotic exploration of unpredictable environments. One such example is the approach, proximity operation, and surface exploration of small bodies. In this article, we present an overview of an estimation framework to approach and land on small bodies as a key functional capability for an autonomous small-body explorer. We use a multi-phase perception/estimation pipeline with interconnected and overlapping measurements and algorithms to characterize and reach the body, from millions of kilometers down to its surface. We consider a notional spacecraft design that operates across all phases from approach to landing and to maneuvering on the surface of the microgravity body. This SmallSat design makes accommodations to simplify autonomous surface operations. The estimation pipeline combines state-of-the-art techniques with new approaches to estimating the target’s unknown properties across all phases. Centroid and light-curve algorithms estimate the body–spacecraft relative trajectory and rotation, respectively, using *a priori* knowledge of the initial relative orbit. A new shape-from-silhouette algorithm estimates the pole (i.e., rotation axis) and the initial visual hull that seeds subsequent feature tracking as the body gets more resolved in the narrow field-of-view imager. Feature tracking refines the pole orientation and shape of the body for estimating initial gravity to enable safe close approach. A coarse-shape reconstruction algorithm is used to identify initial landable regions whose hazardous nature would subsequently be assessed by dense 3D reconstruction. Slope stability, thermal, occlusion, and terra-mechanical hazards would be assessed on densely reconstructed regions and continually refined prior to landing. We simulated a mission scenario for approaching a hypothetical small body whose motion and shape were unknown *a priori*, starting from thousands of kilometers down to 20 km. Results indicate the feasibility of recovering the relative body motion and shape solely relying on onboard measurements and estimates with their associated uncertainties and without human input. Current work continues to mature and characterize the algorithms for the last phases of the estimation framework to land on the surface.

## 1 Introduction

Autonomy is the ability of a system to achieve goals while operating independently of external control (Fong et al., 2018). For a robotic spacecraft, this typically implies operating independent of ground-based control, with limited communication windows and time delays. Autonomy in robotic space exploration is becoming increasingly important to reach and operate in never-visited-before environments, where *a priori* knowledge of that environment has large uncertainties, where the spacecraft’s interaction with that environment is more dynamic in nature, where resources available to the spacecraft are limited, or where the harshness of that environment impacts and degrades the health of the spacecraft. Examples include exploring near, on, or below the surfaces of ocean worlds, such as Europa, Titan, or Enceladus. Even for previously characterized environments, such as the surfaces of the Moon or Mars, autonomy is becoming increasingly critical to improving productivity, increasing robustness, and, eventually, reducing cost, as evidenced by recent advances in autonomous landing and surface navigation for the Mars 2020 mission (Johnson et al., 2015; Toupet et al., 2020) and upcoming mission studies for the planetary science decadal survey^1^, such as the Intrepid lunar mission concept (Robinson et al., 2020).

Small bodies in our solar system include near-Earth objects (NEOs), main-belt asteroids and comets, centaurs, and trans-Neptunium objects such as Kuiper Belt objects. Over 850,000 small bodies have been observed in the solar system, but only 25 have been flown by to date, and as few as seven have been rendezvoused with. The abundance and diversity (both in the composition and the origin) of small bodies drive the need for greater access, which would require more capable and affordable spacecraft (with higher Δ*V*) and autonomous operations to reach, approach, land, move, and sample their surface and subsurface. Increasing the number of accessible small bodies and reducing operational costs are among the key benefits. Greater access enables the study of the population of small bodies. Even exploring a subset of small bodies, such as NEOs, is relevant to multiple thrusts, which are as follows: decadal science, human exploration, *in situ* resource utilization, and planetary defense. Common to the aforementioned thrusts are the following: 1) knowing what is where, 2) characterizing the bodies’ compositions, 3) understanding their geophysical (including geo-technical) properties, and 4) characterizing their environments (Swindle et al., 2019).

NEOs are well-suited targets for advancing autonomy because they embody many of the challenges that would be representative of even more extreme destinations while remaining relatively accessible by small affordable spacecraft (e.g., SmallSats). Technologies developed for autonomously approaching, landing, and exploring NEOs would be applicable to other more remote small bodies. Autonomy is enabling for small-body missions because it would allow greater access to far more diverse bodies than the current ground-in-the-loop exploration paradigm. It enables scaling to missions that would require multiple spacecraft for their measurements, such as bi- and multi-static radar measurements. With onboard situational awareness, autonomy enables closer flybys, maintaining otherwise unstable orbits, maneuvering in proximity to irregular-shaped bodies, and safe landing and relocating on the surface. Approaching small bodies is challenging because of the large-scale changes in the appearance of the target body and the *a priori* unknown motion and shape of the body (mainly for small bodies). Operating near, on, or inside these bodies is challenging because of their largely unknown, highly rugged topographies, their poorly constrained surface properties, and the dynamic nature of the interactions between spacecraft and the bodies due to their low gravity, all of which require autonomy for effective operations. As such, we argue that NEOs are well-suited targets for advancing autonomy, with feed-forward potential to the more challenging outer solar system destinations, including the unknown surfaces of ocean worlds. We argue that autonomy is enabling for small-body missions because it would allow access to more diverse and remote bodies than the ground-in-the-loop exploration paradigm and would scale to missions that require multiple spacecraft^2^. Learning to autonomously reach and explore such environments serves as a stepping stone toward more complex autonomous missions. Once matured, such technologies would have high feed-forward potential to more challenging exploration, such as a Titan aerial explorer that canvasses its terrains, dips into its liquid lakes, or sends probes into its ocean-world interior, an explorer that samples the plumes of Enceladus’ Tiger Stripes, or an explorer that ventures into the crevasses of Europa, to name a few.

The work presented here is a step toward advancing autonomous access to small bodies. The two key contributions of this article are as follows:

• An integrated estimation framework to enable the autonomous approach toward, rendezvous with, and eventual landing on small unexplored bodies. The pipeline leverages a combination of established (centroid and light-curve) algorithms, new algorithms developed by the authors (pole/shape-from-silhouette), and algorithms (feature-tracking, coarse reconstruction, and 3D dense reconstruction) newly applied/evaluated in the integrated pipeline^3^.
• An assessment of the feasibility of the estimation pipeline in maintaining uncertainties below the estimate errors in a realistic mission simulation. This was conducted in a blind experiment that involved approaching and rendezvousing with an unknown, artificially generated body from 3,000 km down to 20 km. The experiment assumes limited *a priori* knowledge of the body with large uncertainties from ground-based astronomical measurements, which included the body’s initial heliocentric orbit, rotation rate, and an approximate pole orientation.

Two secondary contributions that resulted from this investigation are as follows:

• A summary of the results of several trade studies that compared feature trackers and coarse reconstruction algorithms for this domain.
• A notional spacecraft design that can be used to demonstrate an end-to-end autonomous approach, landing, and surface mobility on small bodies.

The autonomy targeted by this work is onboard goal-oriented mission operations. In the European Cooperation for Space Standardization (ECSS), that would correspond to E4 Autonomy (Tipaldi and Glielmo, 2018). While the aforementioned perception-rich estimation pipeline is a key capability for the autonomous approach and landing on small bodies, this functionality would have to be integrated into a system-level capability that would manage intent, activities, resources, and system health (Nesnas et al., 2021). As was pointed out by Tipaldi and Glielmo (2018), there are strong synergies between architectures used for end-to-end autonomous spacecraft and those used in robotics, which have been extensively studied (Alami et al., 1998; Volpe et al., 2001).

This article is organized as follows: in the next section, we summarize key autonomy advances in flight with a focus on small bodies. Section 3 describes the state of the practice in autonomous approach and landing on small bodies, followed by a detailed description of our estimation framework that would enable autonomous approach and landing. In Section 5, we describe our experimental setup, which includes a notional spacecraft design and a high-fidelity simulation of imagery and other telemetry during approach. We summarize the results of simulating a realistic mission scenario for approaching an unexplored body. We conclude with an assessment of the viability of the estimation process in establishing situational awareness and share plans for future development.

## 2 Related Work

Most small-body missions have used some level of autonomy, but all operated within narrow windows and under several constraints, with extensive human-in-the-loop pre- and post-execution of autonomy segments (Nesnas et al., 2021). Autonomy is used in planetary missions either when no ground-in-the-loop alternative exists or when the ensuant actions of an autonomous assessment are nonconsequential from a risk standpoint. In situations where missions had to deploy autonomy, they did so within short-duration windows, with careful ground oversight pre– and post–autonomy deployment, often applying additional constraints informed by prior data and ground-generated models, to restrain the action space of the spacecraft and maintain a comfortable level of predictability of the execution sequence.

### 2.1 Small-Body Cruise and Flyby Operations

In 1999, the Remote Agent Experiment onboard the Deep Space I mission demonstrated goal-directed operations through onboard planning and execution and model-based fault diagnosis and recovery, operating two separate experiments for 2 days and then later for five consecutive days (Bernard et al., 1999; Nayak et al., 1999). The spacecraft demonstrated its ability to respond to high-level goals by generating and executing plans onboard the spacecraft, using model-based fault diagnosis and recovery software. On the same mission, autonomous spacecraft navigation was demonstrated during cruise for 3 months of the 36-month-long mission, executing onboard detection of distant asteroid beacons, updating the spacecraft’s orbit, and planning and executing low-thrust trajectory control. It also executed a 30-min autonomous flyby of a comet, maintaining a lock on the comet’s nucleus as it flew by through updating the comet-relative orbit of the spacecraft and controlling the camera pointing (Bhaskaran et al., 2002).

In the decade to follow, two missions, Stardust and Deep Impact, demonstrated similar feats in tracking comet nucleii on their respective missions to three separate comets (Bhaskaran, 2012). Furthermore, in 2005, the Deep Impact mission performed the most challenging use of autonomous navigation to date (Kubitschek et al., 2006). The onboard system guided the impactor spacecraft during the final 2 h to collide with the 7-km-wide comet Tempel 1 at a speed of 10.1 km/s. Although not the purpose of the mission, it was the first to show that a kinetic impactor could be used to deflect a potentially hazardous asteroid from hitting the Earth using the same technology. Most recently, the ASTERIA spacecraft transitioned its commanding from time-based sequences to task networks and demonstrated onboard orbit determination in low Earth orbit (LEO) without GPS, using passive imaging for orbit determination (Fesq et al., 2019).

### 2.2 Small-Body Proximity Operations

Operating in proximity to and on small bodies has proven particularly time-consuming and challenging. To date, only five missions have attempted to operate for extended periods of time in close proximity to such small bodies: NEAR Shoemaker, Rosetta, Hayabusa, Hayabusa2, and OSIRIS-REx (Yeomans et al., 2000; Herfort and Casas, 2015; Lorenz et al., 2017; DellaGiustina et al., 2019; Kitazato et al., 2019; Ogawa et al., 2020). Many factors make operating around small bodies challenging: the microgravity of such bodies, rocks and debris that can be lifted off their surfaces, outgassing events, their irregular topography and correspondent sharp shadows and occlusions, and their poorly constrained surface properties. The difficulties of reaching the surface, collecting samples, and returning these samples stem from uncertainties of the unknown environment and the dynamic interaction with a low-gravity body. The deployment and access to the surface by Hayabusa’s MINERVA (Yoshimitsu et al., 2012) and Rosetta’s Philae (Biele and Ulamec, 2008) highlight some of these challenges and, together with OSIRIS-REx, underscore our limited knowledge of the surface properties. Because of the uncertainty associated with such knowledge, missions to small bodies typically rely on some degree of autonomy.

In 2005, the Hayabusa mission demonstrated autonomous terminal descent of the last 50 m toward a near-surface goal for sample collection, using laser ranging (at <100 m) to adjust altitude and attitude (Kubota et al., 2012). This capability was also employed on the 2019 Hayabusa2 mission, where the mission used a hybrid ground/onboard terminal descent with the ground controlling the boresight approach, while the onboard system controlled the lateral motion for the final 50 m. In 2020, the OSIRIS-REx mission used terrain-relative navigation for its touch-and-go maneuver for sample acquisition (Lorenz et al., 2017). Using a ground-generated shape-model, the spacecraft matched natural features to images rendered from the generated model to guide the spacecraft in the last several hours of descent to reach a precise spot for touch-and-go sampling. This segment was executed autonomously but with considerable pre-planning and testing to ensure safety. In 2022, the planetary defense mission DART is planning to use autonomous targeting to impact the 100-m secondary body Dimorphos of the Didymos binary asteroid system, perturbing the secondary body’s orbit adequately to be observed using ground telescopes (Cheng et al., 2018). During its terminal approach, the spacecraft will use onboard perception to distinguish between the two bodies, lock onto the secondary body, and guide the spacecraft toward it.

For science exploration, surface interaction is needed for microscopy, seisomology, and sampling. The OSIRIS-REx mission captured samples from the surface of the asteroid Bennu using a 3.4-m extended robotic arm in a touch-and-go maneuver that penetrated to a depth of 50 cm, well beyond the expected depth for the sample capture, an indication of poorly constrained surface properties. In addition to sampling, mobility on the surface (or spacecraft relocation) could greatly enhance the value of a landed system by exploring distinct regions on the body (Castillo-Rogez et al., 2012). Proximity and surface operations, in particular, ones that have additional orientation constraints for thermal, power, or communication reasons, require six–degree-of-freedom (DOF) autonomous guidance, navigation, and control (Riedel et al., 2008; Bayard, 2009; Hockman et al., 2017). Such capabilities include perception, feature tracking for motion estimation, 3D mapping, hazard assessment, motion planning, and six-DOF control. These autonomy-enabling functions require a system-level executive (Grasso, 2002; Verma et al., 2006; Fesq et al., 2019) to orchestrate these functions with the planning, execution, system health management, and data management, as was demonstrated in prior studies involving landings on the Moon and on small bodies (Riedel et al., 2008; Riedel et al., 2010), and for understanding fundamental trade-offs concerning small-body touch-and-go sampling (Cangahuala et al., 2011).

Autonomous spacecraft operations, especially in the vicinity of unexplored bodies and surfaces, are particularly challenging despite our best efforts to anticipate the execution of pre-scripted sequences. The unpredictability of the outcome drives the need for *in situ* situational awareness and reasoning for subsequent actions. As technologies in sensing, computing, and reasoning mature, the viability of autonomous operations could be realized to a much larger degree in the coming decades. Consequently, the state of the practice in accessing small bodies remains largely driven by a ground-operation team of scientists and engineers, employing autonomous operations only in critical phases that cannot afford the constraints of ground-in-the-loop decision-making. Developing and sharing of the capability that enables autonomous approach, landing, and surface operations on small bodies would allow the maturing of autonomous spacecraft capabilities needed for more extreme destinations, and it would allow scaling in both the number of missions and the number of spacecraft that can explore the large number of small bodies.

## 3 State of the Practice

The current state of the practice for approaching and landing on a small body, from first detection by the spacecraft to landing, is heavily dependent on ground-in-the-loop operations. Despite the use of autonomous functions, and in some cases, repeated use of such functions^4^, all missions to date were primarily executed in a manner where command sequences are uploaded and executed in lock step with ground-planning cycles. The process begins with deep space navigation, which relies heavily on radiometric spacecraft tracking using a network of ground antennae (e.g., the Deep Space Network (DSN)). The remarkable accuracy of radiometric measurements has been the hallmark of deep space navigation, providing line-of-sight radial ranging accuracy on the order of a few meters and Doppler-based line-of-sight velocity measurements on the order of tenths of millimeters per second. For approaching and rendezvousing with bodies whose orbits are highly uncertain (including asteroids, comets, planetary satellites, and trans-Neptunian objects), optical navigation (using an onboard camera to image the body) is used as it provides a target-relative measurement. The data are typically processed on the ground and combined with the radiometric data, except for the aforementioned missions, which performed specific phases autonomously onboard.

Specifically for encounters with small bodies, the approach phase begins with the first detection of the body, typically several months prior to the encounter, at distances in the high tens of millions of kilometers. In this phase, the body is almost always unresolved and simple center-finding techniques are used to precisely locate the center of brightness. This information, in conjunction with ground-based radiometric data, is used to improve the ephemeris of the target body, which, up to this point, is based entirely on observations from the Earth. The Earth-based knowledge of the body’s orbit can be anywhere, from fairly accurate (in the low tens of kilometers) in the case of larger well-known asteroids to extremely poor (many thousands of kilometers) in the case of small, dim asteroids and comets (due to large and unknown outgassing accelerations acting on them). As the spacecraft continues its approach, trajectory correction maneuvers are implemented using the refined knowledge of the target-body ephemeris to specifically target either a flyby or a rendezvous location. In addition, analysis of light-curve information from the body can be used to update its spin rate (periodicity).

As the spacecraft gets closer and the body becomes resolved, additional information can be gleaned from the data. By tracking features on the surface, information about the body’s orientation in space, including the direction of the pole, as well as determining whether the body is rotating around a principal axis or tumbling, can be computed. The current and most widely used method to identify and track surface features is called stereo photoclinometry (SPC) (Gaskell et al., 2008). SPC relies on using multiple images of the body to build detailed maplets of the surface, simultaneously solving for both the topography and the albedo in a least-squares fit. The method uses *a priori* information of the spacecraft’s position and attitude to create the network of maplets in body-fixed coordinates used for tracking features. The process becomes iterative; as surface features are precisely located on the surface, it can be used to refine the position of the spacecraft, which, in turn, can be used to improve the features of the surface location, and so on. This process continues throughout the proximity operation phases to constantly refine the spacecraft’s orbit, the body-fixed surface-feature locations, and information about the pole orientation and spin rate of the body. The refined orbit estimates, which include merging radiometric and optical data, are also used to plan maneuvers for proximity operations, including landing, if needed. Finally, the orbit estimates also improve knowledge of the body’s overall mass and gravity field.

Although this ground-based methodology has proved very successful on multiple missions, including Dawn, Rosetta, and OSIRIS-REx, it is very labor-intensive, requiring large operations teams for extended durations. By necessity, closing the loop between receiving navigation measurements and sending control commands for maneuvers or pointing instruments is limited by, at a minimum, the round-trip flight time to the spacecraft, but also the time it takes to process the large volume of data and generate the ground-based plan.

With the increase in sensing, computation, and algorithms, mature autonomous systems could significantly reduce the operational duration of the proximity and surface phases and increase their productivity. An autonomous spacecraft would always use the most recent information and reason about and reconcile that information onboard to establish situational awareness across the approach through the landing phases to guide the spacecraft’s actions. All the processes currently performed on the ground, that is, estimating the spacecraft’s orbit relative to the target and the body’s spin rate, pole orientation, and coarse shape, need to be transferred to the spacecraft. The spacecraft then needs to assess all hazards associated with proximal interactions, landing, and surface mobility, if planned. Such hazards arise from topography, rock types and distribution, ejecta/plume, surface stability (slope and sinkage), line-of-sight occlusions, and thermal traps (hot/cold).

## 4 Approach

### 4.1 Assumptions

The problem of changing the paradigm from ground-in-the-loop to autonomously approaching and landing on a small body, including operating on the surface of the microgravity body, is both compelling and challenging. The large-scale changes perceived across millions of kilometers, the sharp shadows imposed by a rugged airless body, the *a priori* unknown and irregular shape of the body, the rotation and nutation of the body, and the impact of an irregular gravity field on the spacecraft all but make this problem intractable.

To advance the art, we list the assumptions for our autonomous estimation framework.

• Principal-Axis Body Rotation: We assume that the body primarily has a principal-axis rotation without substantial nutation (i.e., the body is not tumbling). As expected, the vast majority of asteroids are found to have a uniform rotation around their maximum moment of inertia, as it is the minimum energy rotation state of a body (Burns and Safronov, 1973). To date, this assumption held for the bodies that spacecraft have rendezvoused with (Itokawa, 67P, Eros, Ryugu, and Bennu). However, on the small-size scale, there is a set of fast-spinning, tumbling bodies that do not currently have a clear theoretical explanation for their existence or persistence (Scheeres, 2016). These bodies would be challenging to rendezvous with. We will address adaptations of this estimation pipeline to such bodies in future work.
• Small Sun-Phase Angle Approach: We assume an approach with a relatively small angle (<20°) off the sun-body line with the Sun behind the spacecraft and the target body. This allows the body to be nearly fully illuminated from the spacecraft’s vantage point, minimizing major shadowed regions of the body. This is justified because the approach can be controlled as part of the planned trajectory and is commonly used in practice. Our team is working to relax this assumption.
• Availability of an initial low-fidelity relative orbit: We assume the availability of initial estimates with large uncertainties of the target-relative orbit based on navigating the spacecraft to this particular point. This is usually available from ground-based observations.
• Availability of spacecraft attitude knowledge: We assume that the spacecraft attitude knowledge is available throughout the approach and landing phases using standard star-tracking techniques. Multiple measurement sources inform the spacecraft attitude in addition to the star tracker: the target-body location in both the narrow- and wide-FOV cameras as well as inertial sensing (in the relative sense only), which are fused onboard using an attitude estimation algorithm. However, in situations where the spacecraft loses attitude knowledge, which can sometimes occur in safing events, there are procedures to recover attitude, such as putting the spacecraft into a sun-cone mode, where the panels are on the Sun to maintain power, but the spacecraft spins to maintain stability without attitude knowledge. Once the spacecraft regains its three-axis stability from the star trackers, it can resume the execution of the estimation pipeline. The estimation pipeline does not impose requirements on maintaining a view of the target throughout the approach and landing (i.e., strict target-pointing constraints). However, we designed our notional platform to have a great degree of “attitude flexibility” to limit such constraints (see Section 5.1 for more details).

### 4.2 Multi-Phase Estimation Framework

**FIGURE 1:**
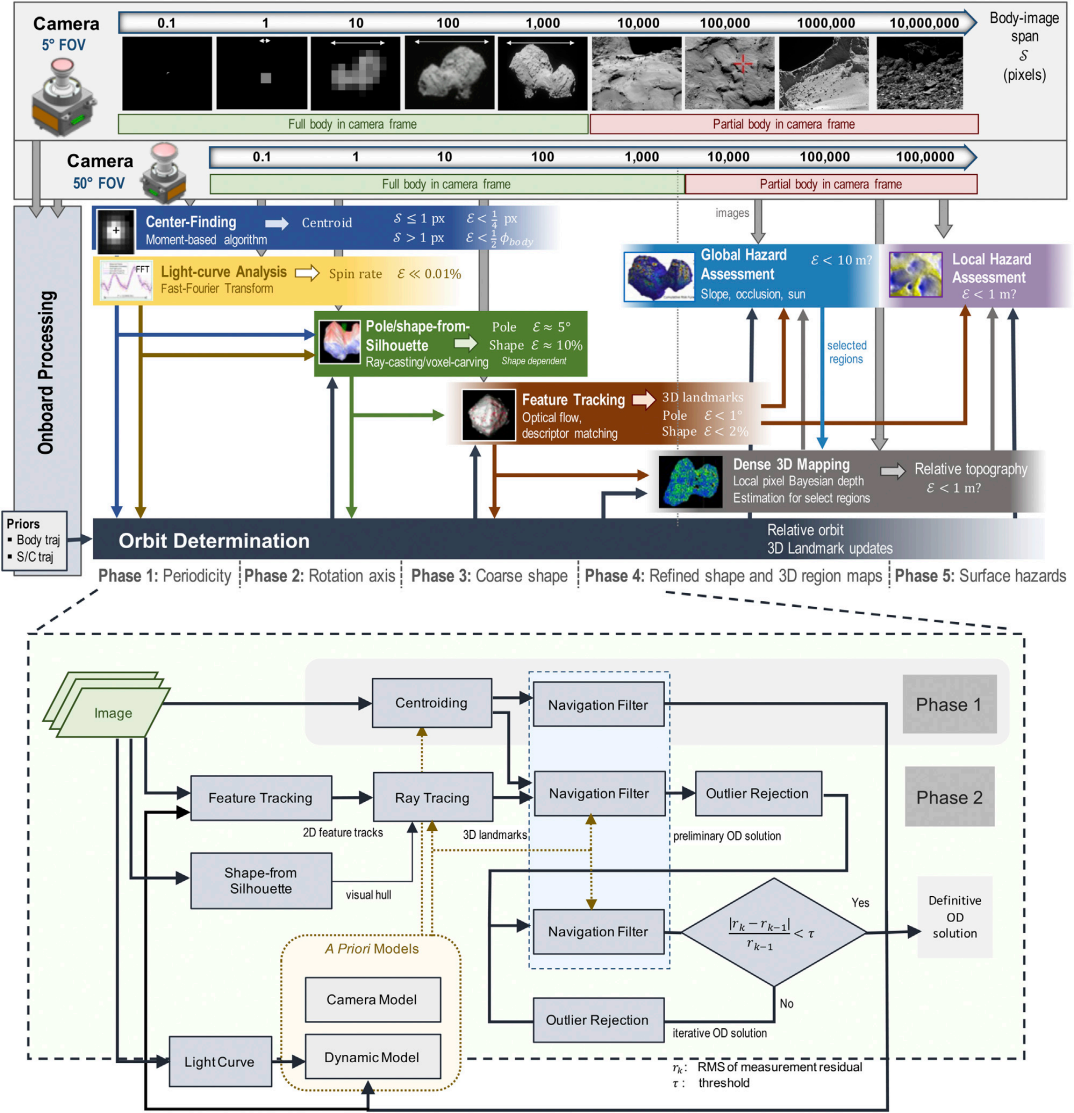
Multi-phase single-spacecraft and target body estimation framework **(A)** with details of the autonomous orbit determination process **(B)**.

To enable the autonomous approach, rendezvous with, and safe landing on a small body, we developed the multi-phase estimation framework (Figure 1) that relies on onboard optical and inertial sensing. At the start of the approach, the framework is seeded with prior orbit estimates and associated uncertainties from navigation results up to that point. The framework then uses one or two parallel pipelines, one for each of its two boresighted, radiometrically calibrated cameras. The narrow-FOV camera, typically 5°–10°, is used for distant approach, while the wide-FOV camera, an order of magnitude wider, is used for close approach, landing, and surface mobility. The two cameras allow for staggering the estimation pipelines by an order of magnitude in the body-image span and generate redundant measurements. The body-image span denotes the maximum width in pixels that the body subtends in the image at a given distance, which is a scale-invariant metric of body size, body distance, and camera optics that we use to inform transitions across phases. The overlap in the sensing and estimation across the phases provides multiple sources of knowledge and redundant measurements that have to be reconciled to establish robust situational awareness. The autonomous situational awareness pipeline consists of five phases that estimate the following: the 1) spin rate (periodicity), 2) pole orientation (rotation axis), 3) initial shape, 4) refined shape and 3D mapping, and 5) hazard assessment for landing. In addition, orbit determination is performed continuously throughout all phases.

### 4.3 Determining Relative Orbits

To determine the trajectory of the spacecraft relative to the target and other parameters of interest (e.g., the target’s orientation and gravity parameters), we use a standard batch state-estimation algorithm that is informed by optical-based measurements (both centroid measurements and feature tracks when the target is sufficiently resolved) and any available *a priori* information (e.g., maneuver models). Estimation of the spacecraft’s orbit parameters (i.e., orbit determination or OD) is a continuous process from initial acquisition of the body after launch until the spacecraft completes its orbital operations, but the process becomes target-relative once the body becomes detectable in images during the early stages of approach.

With only centroid measurements, the relative orbit begins as weakly observable due to a lack of information concerning the system’s scale (i.e., spacecraft distance to the target and the size of the target); however, as the data arc becomes longer, the combination of information from the measurements and the state dynamical model yields a complete estimate of the spacecraft’s relative orbit^5^. Estimation of the system’s scale can be specifically improved in the presence of well-modeled maneuvers. Uncertainty will generally be largest in the boresight direction due to the nature of the optical measurements.

When the body-image span becomes large enough to resolve surface features, feature tracks are fed into the state-estimation algorithm. A feature is the projection of a surface landmark onto the camera’s 2D plane, and a landmark is its corresponding 3D location on the surface relative to the body’s inertial frame. Feature tracks enable the estimation of the target’s orientation (i.e., the spin rate and pole) and the scale of the body (*via* the estimated feature positions). To help ensure that an accurate solution is being computed, we run multiple filter variations in a filter bank, where the solutions can be compared against one another in order to check for solution consistency. Filter-bank variations include the types of measurements used in each cycle (i.e., whether centroids, tracked features, or both types are used) and landmark modes used (i.e., in our case, whether a drift model for landmarks is used or not). Solutions that differ in a statistically significant manner indicate an anomaly in the modeling of one or more filters. This filter-bank approach is important to ensuring successful transitions across phases, from centroid-only solutions to feature-only solutions, which is necessitated when the body becomes too large in the image plane to support the extraction of an accurate centroid measurement. This phase transition is also critical for effective close-proximity operations around the target, which is a precursor to landing. Figure 1B summarizes the orbit-determination process that combines estimates of the spin, pole, visual feature tracks, initial visual hull, and models of the dynamics and the camera(s) to estimate the body’s motion and its 3D landmarks.

In a batch state-estimation process, there is a computational limit on the size of each data arc (which is the combination of the length of the arc and the number of features therein). Shorter data arcs allow for a larger number of features to be included, but they also reduce the robustness of the estimation, especially in the presence of biased or statistically anomalous data. To stay within the computational limit, we use a feed-forward approach to balance the length of the arc with the number of features (down-selecting to higher-quality, persistent, and geometrically distributed features/landmarks). In this approach, we use data arc priors (estimates and associated uncertainties) to properly constrain the solution and increase robustness. Geometrically diverse landmarks help preserve the orientation and scale information of the full feature set.

While landmarks are typically fixed in the body’s inertial frame, feature drift in the image impacts the estimates of landmark locations on the body’s surface. We model statistically significant drift, such as the one resulting from moving shadows. Our primary drift model estimates a constant angular velocity in the target’s body-fixed frame for each landmark. This angular velocity is expected to be small relative to the rotation rate of the body, but the model helps remove deterministic biasing associated with feature drift. An alternate model estimates the drift as a translational velocity; however, this model suffers from strong correlations with the spacecraft’s impulsive maneuvers, which can strongly degrade filter performance in some situations.

### 4.4 Estimating the Body Centroid

The first closed-loop measurement with the target body is that of the body’s centroid, which can be used as soon as the body is detected in the narrow-FOV camera. Depending on the size of the body and the camera optics, the spacecraft would typically be tens of millions of kilometers away from the body. Because of the camera optics, the reflected light from the sub-pixel–sized body would appear as a point-spread function across several pixels in the focal plane. By setting the proper camera exposure, both the target body and the background star field would be observed. A centroid brightness-moment algorithm (Owen, 2011) is applied to search windows around the predicted target and star locations based on ephemeris data to estimate the body and stars’ initial centroids. The window sizes are typically 10 × 10 to ∼100 × 100 pixels. A Gaussian function is then fit to the initial target location and each star location in the image, resulting in an accuracy of 0.01–0.05 pixels for the stars and ∼0.1 pixels for the body (Bhaskaran, 2012).

As the body’s signature in the image grows, the brightness-moment algorithm (Owen, 2011) gets used without the Gaussian fit. Estimates of body centroids are subsequently used as priors for the center of mass in orbit determination. In most cases, the center of brightness does not represent the center of rotation as the target body is unlikely to be uniform in shape and density. As the body size grows in the image, the accuracy of the brightness centroid decreases, and the pipeline transitions to relying more on feature tracking to estimate the pole center.

### 4.5 Estimating Periodicity (Spin Rate)

Estimating the spin rate (i.e., “periodicity”) of the target body can start as soon as the body is detected and continues until surface features get resolved in the images and feature-based orbit determination generates stable results. While in theory, one can continue to estimate periodicity as long as the full extent of the body remains visible in the camera’s frame, there are diminishing returns and negative effects as the body grows in the image, such as perspective distortion and non-collimated light from the body. For targets that have been sufficiently observed from ground telescopes prior to launch, an initial estimate of the spin rate may exist. For other targets, such as comets, the spin rates may not be well constrained by ground observations because of the difficulty in discriminating between internal light reflection within their coma and variations in reflected light from their surfaces (Scheeres, 2016). In either case, onboard algorithms would be needed for validating and/or refining estimates of the spin rate to a sufficient accuracy for subsequent algorithms within the autonomous approach pipeline. During distant approach, the spacecraft has plenty of time to capture many images over several rotations to refine the light curve estimates.

The principle of onboard period estimation is based on the well-established technique of light-curve analysis (refer to Warner et al. (2009) for a more detailed description), which extracts the principal period of fluctuating intensity of the reflected light from a rotating body. Advanced light-curve inversion techniques have also been used to estimate crude shapes of the body (Kaasalainen et al., 2001). Various algorithmic techniques have been used to extract the principal period of rotation from a light curve, including simple “peak detection,” Fourier transforms, and regression methods (Waszczak et al., 2015). We adopt the robust and highly accurate Fourier regression method, in which an *n*th order Fourier series is fit to the time-series intensity, *δ*(*τ*), the data being as follows:

$$\delta \left(\tau \right)=A_{0}+\overset{n}{\underset{k=1}{\sum }}A_{1,k}\sin\left(\frac{2\pi k\tau }{P}\right)+A_{2,k}\cos\left(\frac{2\pi k\tau }{P}\right),$$ (1)

where *P* is the principal (first harmonic) period and *A*_*i*,*k*_ are coefficients to be fit. Since this functional form requires zero-mean fluctuations with a constant bias and amplitude, in practice, when fitting data over a longer time-series, other correcting terms may be included to remove effects such as the increasing brightness or the varying observation phase. Also, due to the bilobate nature of asteroid shapes, the first harmonic period of a Fourier fit can sometimes correspond to twice the true rotation period, but simple logical checks can be used to detect and correct for this (see Warner et al. (2009) for more details).

**FIGURE 2:**
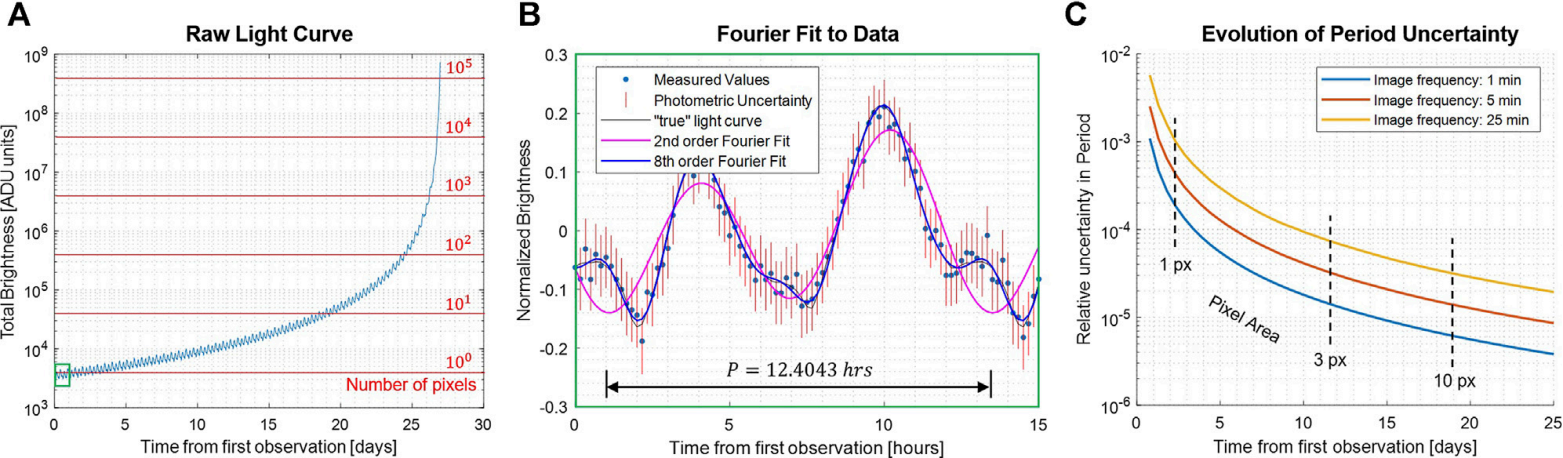
Example of a light-curve analysis for a simulated radial approach of comet 67P, starting from 35,000 km and approaching at 15 m/s with a 5^*o*^ solar phase angle and a Hapke reflectance model (Hapke, 2012). **(A)** Raw light curve, showing increasing brightness during approach. **(B)** Periodicity estimation by Fourier regression, showing raw data (normalized), error bars, and two different-order Fourier models. **(C)** Evolution of relative-period uncertainty over time for three different imaging frequencies.

Figure 2 shows an example of the period estimation process for a simulated approach of comet 67P. The left plot illustrates how the increasing mean brightness must be corrected for long integrations. The center plot illustrates how different-order Fourier models may be used to better fit the data. In general, higher-order Fourier models can produce a much better fit to the light-curve data (and more accurate period estimates) but require more data and computation. A 2^nd^-order fit is generally more robust and sufficient for capturing the first harmonic. Finally, the right plot illustrates the decay of period uncertainty as the spacecraft approaches the body for different imaging frequencies. In practice, with autonomous image processing, image frequencies may be well below 1 min, generating estimates with a relative error in the order of 10^–5^ (e.g., less than 1 s in a 12-h period) by the time images are sufficiently resolved for subsequent silhouette analysis.

This technique relies on the existence of shape–albedo asymmetries in the body, which tend to be significant for small asteroids. For example, highly irregular bodies like Itokawa can fluctuate in magnitude by over 100%, but even nearly cylindrically symmetric bodies like Bennu and Ryugu tend to vary by more than 10% due to local variations in topography. Thus, the exact distance at which a robust light curve can be estimated depends on the size and asymmetry of the body and the “photometric capabilities” of the camera; but generally, we have found that the period can be estimated with sufficient accuracy for subsequent algorithms when the body is only a few pixels (or less) in the body-image span.

### 4.6 Estimating the Pole Orientation (Axis of Rotation)

For bodies that have a stable spin around their principal axis of inertia, their pose evolution is entirely given by the pole orientation and the spin rate, as the body’s prime meridian (Owen, 2011) (PM) can be arbitrarily defined. Since the uncertainty in the spin rate from Phase 1 is sufficiently small^6^, this problem is reduced to estimating the pole orientation, which is defined by the angles of right ascension (*α*) and declination (*δ*) (RA and Dec). We start estimating the pole orientation when the body spans tens of pixels, before distinct surface landmarks are sufficiently resolved in the image. We use the body’s silhouette to both estimate the pole and construct an initial shape.

Shape-from-silhouette techniques have been extensively studied in the computer vision community and have been used to extract shapes from images and video streams (Laurentini, 1994; Cheung et al., 2005a; Cheung et al., 2005b). They have also been used in ground-based spacecraft operations (e.g., during the Rosetta mission (Lauer et al., 2012; de Santayana and Lauer, 2015)). However, using the silhouette also has some limitations. First, the resultant shape, known as the visual hull, does not represent the true shape of the body as it cannot observe two-dimensional concavities, such as craters (Laurentini, 1994). Rather, it is the maximal shape encompassing the true shape of the body. Second, there is an inherent ambiguity in estimating the pole of a spinning object, which results from the bi-stable illusion of two likely pole orientations for any given set of silhouettes: one being the true pole and the other being an illusory pole that is a reflection of the true pole across the observation axis. This illusion, for which humans are biased to interpret the pole rotation as an above-equator, clockwise pole rotation, is known as the “ballerina effect” (Troje and McAdam, 2010). Third, the use of silhouettes degenerates when the observation axis aligns with the body’s rotation pole and the body nears axisymmetry around that pole, since changes in the observed silhouette of the body diminish as the body rotates. Despite these limitations, the strong signature of the body’s silhouette against the dark sky, together with its stable rotation, provides valuable information to establish and constrain knowledge of the body’s rotation and shape at large distances. Ambiguities and degeneracy, should they occur, would be resolved using subsequent observation geometries and/or in the target-tracking phase of the pipeline.

To find the pole using the body’s silhouette, we use a multiple-hypotheses ray-casting method that searches the (*α*, *δ*) domain for the most likely pole. In this method, a 3D ray is cast from the camera to each pixel of the body using the estimates of the pole center and the distance from the camera to the body. For a given hypothesis of pole angles (*α*_*i*_, *δ*_*j*_) in the search domain, a 3D ray for each body pixel is rotated from its initial observation to a subsequent one, using the rotation rate and times of observations. The rotated ray is then projected back onto the camera and checked against the new observation for consistency. Re-projected rays that do not intersect the body in the newly observed image are discarded. The process is repeated for each body pixel and through its full rotation. After a full rotation, the re-projected silhouette, formed by the intersecting viewing cones, is compared to the observed silhouette for overlap. Eq. 2 defines the overlap error metric, *ϵ*, between the silhouette of the projected visual hull based on the pole hypothesis ( $S_{h}$ ) and the observed silhouette ( $S_{0}$ ), where A denotes the area in pixels.

$$\epsilon \left(S_{h},S_{0}\right)=\frac{A\left(S_{0}\cup S_{h}\right)-A\left(S_{0}\cap S_{h}\right)}{A\left(S_{0}\right)}$$ (2)

As the hypothesized pole converges toward the true pole, the overlapping error, *ϵ*, goes to zero. We claim that the likelihood of the pole is correlated with the overlap error. We define a heuristic for pole likelihood as shown in Eq. 3, where L is the pole-estimate likelihood function.

$$L\left(\alpha ,\delta \right)∼\frac{1}{\epsilon \left(S_{h},S_{0}\right)}$$ (3)

To identify viable poles, we use a maximum-likelihood-estimation approach to locate global maximal regions and subsequently estimate their corresponding local maximum. These pole hypotheses are used as priors in the orbit determination process, which refines the pole estimates and helps disambiguate illusory ones. We performed sensitivity analyses with respect to the sun-phase angle (i.e., the angle between the sun–body line and the spacecraft–body line^7^). Results show that the algorithm can accurately estimate the pole at sun-phase angles up to ±90° when approaching the body at zero latitude. Ongoing work is underway to analytically formulate the relation between the overlap error and pole likelihood and to characterize the uncertainty of pole estimates at different latitudes. More details on this algorithm can be found in the study by Bandyopadhyay et al. (2021). This method estimates the pole without unnecessarily generating multiple visual hulls. As such, it is a more efficient alternative than our original method that generated a visual hull for each hypothesized pole orientation (Bandyopadhyay et al., 2019; Villa et al., 2020).

### 4.7 Estimating the Initial Shape (Visual Hull)

As a byproduct of the silhouette-based pole-estimation algorithm, we extract a global shape model by carving out those volume elements (voxels), from an initial volume, which do not fit within the set of body silhouettes as seen from the estimated camera poses. By the nature of its construction, the visual hull does not represent the true shape of the body, as previously discussed. Nonetheless, it still presents a useful bounding volume for subsequent shape modeling, an approximate surface on which to project image features to estimate their 3D landmarks, and may indeed be close to the true shape, if the body is largely convex. The quality of the observed visual hull greatly depends on the relative orientations of the Sun, the spacecraft, and the pole (i.e., the phase angle and observation axis) and, as such, is susceptible to the aforementioned degenerate conditions. Large distortions can occur if the pole is highly inclined with respect to the Sun or the spacecraft. For example, in the limit as the pole, Sun, and observation axes align^8^, only half of the body receives sunlight and is ever observed. As such, only the equatorial silhouette of the body is visible, leaving the visual hull in the orthogonal direction (the pole direction) completely unconstrained. Thus, care must be taken in the interpretation of the visual hull as a proxy for the body shape. By controlling the approach vector and, hence, varying the observer’s latitude, some degeneracy can be mitigated.

Forbes has shown that a discrete number of silhouette observations yield a finite error in the visual hull estimation and decrease with improved vantage point coverage (Forbes, 2007). In this application, the error in the visual hull of the rotating body is constrained by the body-image span and the frequency of imaging. Onboard autonomy enables more frequent imaging and processing, only constrained by available computing time but not by communication constraints. This enables an arbitrarily low visual hull estimation error, up to the image pixelation limits. But even at large distances where the body spans fewer pixels, higher-resolution visual hulls could be constructed using sub-pixel silhouette techniques. Eq. 4 shows the relation between the visual hull estimation error and the sampling frequency, where *D*_*px*_ is the body diameter in pixels, *ω*_*B*_ is its spin (rotation) rate, and *δ*_*px*_ is the maximum visual hull estimation error, in pixels. *K*_*S*_ is a correction factor to account for the body’s non-sphericity (*K*_*S*_ = 1 for a sphere, otherwise *K*_*S*_ < 1). Here, we chose *K*_*S*_ = 0.25. Changes in the approach latitude and body-apparent size are considered negligible over a single rotation-period time frame. This equation can be used as a guideline (or as an actual control law) for the appropriate imaging frequency for the autonomous system.

$$f_{S}=\frac{\omega_{B}}{2\cos^{-1}\left(D_{px}/\left(D_{px}+2K_{S}\delta_{px}\right)\right)}$$ (4)

Once we identified the pole (or multiple candidate poles), we use the voxel-carving algorithm defined by Forbes (2007) and Bandyopadhyay et al. (2019) to generate an initial visual hull. Voxels are volumetric elements organized in a three-dimensional grid that represent a discretized shape. Multi-resolution voxels can be used to reduce the memory footprint. Voxels projected onto the camera plane that lie outside the body’s silhouette in all images of a full rotation are carved out. To resolve the status of voxels belonging to the image foreground only in some of the images, an octree-based search is performed. Voxel size is adaptive and increases with image resolution.

**FIGURE 3:**
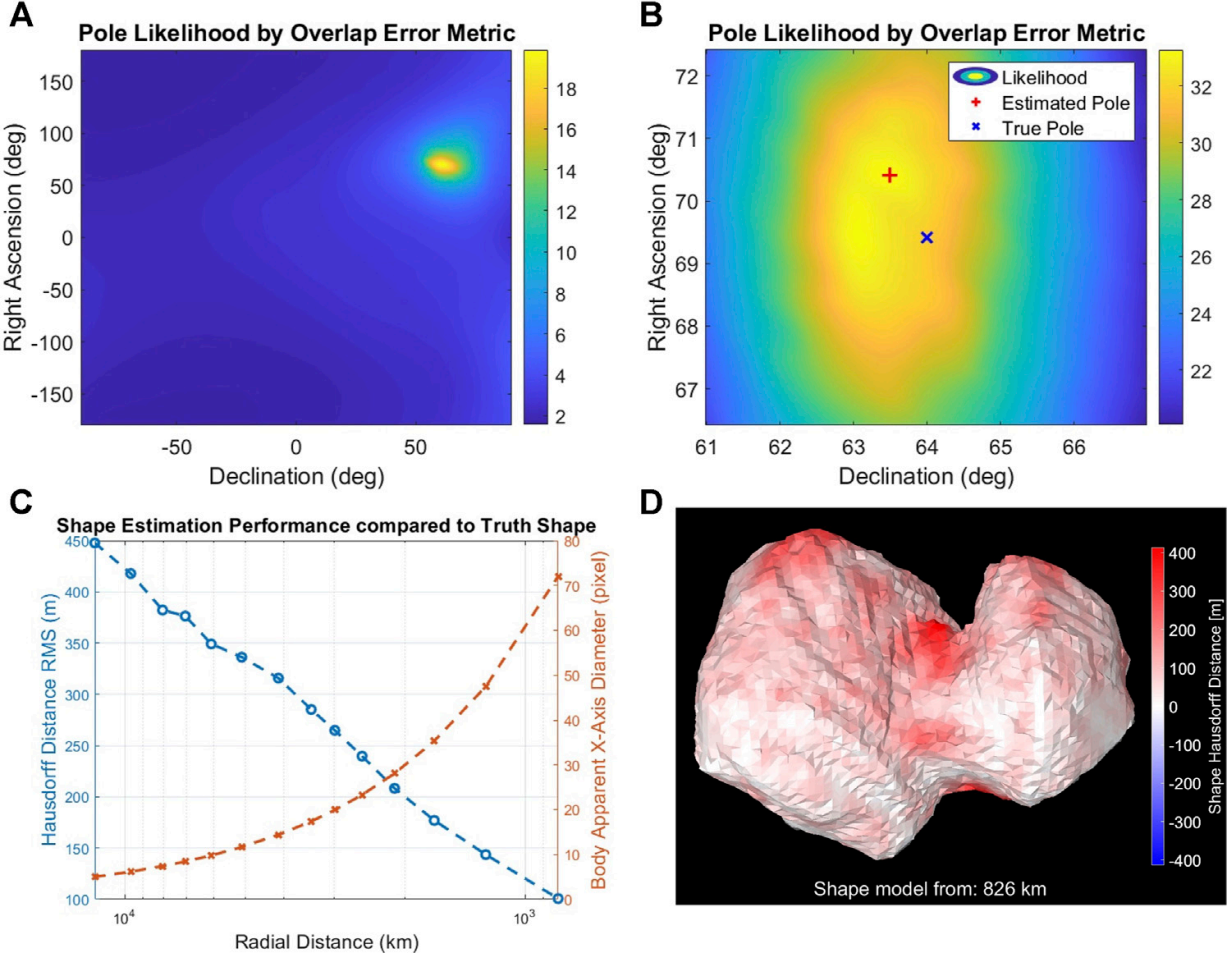
Pole likelihood evaluated at 10° **(A)** and 0.5° **(B)** intervals at a distance of 826 km from the body. **(C,D)** Performance of shape-from-silhouette as a function of the body-image span **(D)** and errors in the reconstructed visual hull (i.e., Hausdorff distance) at 826 km.

Figure 3 shows the results of estimating the pole and the visual hull from simulated images of comet 67P. We empirically verified that at an apparent 55-pixel body-image span, for a global search resolution of (Δ*α* = 10°, Δ*δ* = 10°) followed by a 0.5° regional resolution within ±3° of the coarse resolution peak, the pole accuracy was within 2° from the true pole. To characterize the accuracy of the reconstructed visual hull at different distances/resolutions, we reproduced the Rosetta spacecraft trajectory, simulating more frequent images of 67P using high-fidelity rendering (Section 5.2) with a nonzero sun-phase angle for the entire approach (Castellini et al., 2015). We constructed independent visual hulls at 20 intervals from 10^4^–10^3^ km, each covering a single rotation imaged at the pixelation limit frequency, *f*_*S*_. The error between the reconstructed shape and the ground truth uses the mean Hausdorff distance (Oniga and Chirila, 2013). The Hausdorff distances are the minimum distance between the reconstructed vertices and those of the ground-truth model. The mean Hausdorff distance is a widely used metric for reconstruction accuracy. Normalizing by the ground-truth diameter gives a dimensionless parameter for comparison across different bodies. Figures 3C,D show the monotonically decreasing root mean square error of the Hausdorff distance relative to the true shape at 826km. As expected, the nonobservable concavities show the largest errors in dark red.

### 4.8 Feature Tracking

This phase starts when surface landmarks become sufficiently resolved in the camera image to be distinguishable from one another and trackable over multiple frames. Recall that features are the 2D image reprojection of their corresponding 3D landmarks. Feature tracking has been extensively studied in the computer vision and robotics communities, resulting in the maturation of several feature-extraction approaches (corner detectors and feature descriptors), followed by either optical flow tracking or descriptor matching. Both classical machine vision and machine learning techniques have been used extensively in terrestrial applications, with a few that have been used in space applications.

For this application, salient visual features are tracked across multiple images, and their tracks in the image planes are used for orbit determination (see Section 4.3) to refine estimates of the body’s motion parameters (relative trajectory and rotation) as outlined in Figure 1B. Using the camera model and distance estimates, 2D features are ray-traced to the visual hull to generate an initial set of corresponding landmarks. Then, using estimates of the spin rate, pole orientation, initial visual hull, visual feature tracks, and their initial corresponding 3D locations as input, together with models of the dynamics and camera model(s), the orbit determination filter updates the body-relative motion parameters and feature/landmark locations. Updated post-fit estimates are used to identify and remove outliers, and the process is repeated until the residuals are within a user-set tolerance. The orbit determination and landmark refinement process continue from this point onward until landing.

Critical to feature tracking is the quality of the visual features. Their ideal characteristics for orbit determination are as follows: 1) they can be tracked over many observations, 2) they can be recovered after a full-body rotation, following being eclipsed by the body during the second half of the rotation (a.k.a. loop closure), 3) they have a low positional error, 4) they have a Gaussian error, 5) they have few outliers (features with a large positional error), and 6) they are evenly distributed across the body. Achieving these characteristics is exceedingly difficult for small bodies because of the following:

1) lighting changes that cause dramatic changes in visual appearance as the body rotates and which are accentuated by the absence of an atmosphere to diffuse light,
2) low-contrast features from typically low-albedo bodies,
3) self-similar features from an often consistent albedo,
4) concentrated features on the body that occupies only a small fraction of an image; the concentrated features on the body represent a narrow view-port surrounding the spacecraft, making geometry-based outlier rejection more challenging, and
5) large-scale changes from the start of the approach down to the surface.

Despite their substantial maturity for Earth-based applications, feature-tracking algorithms face specific challenges for small bodies, which warrant further examination. In the study by Morrell (2020), we compared state-of-the-art open-source feature-tracking algorithms, which included an optical-flow approach (KLT with Shi–Tomasi features (Tomasi and Kanade, 1991)), to feature-descriptor approaches ranging from local histograms (scale invariant feature transforms; SIFT (Lowe, 1999)), to wavelets (speeded up robust feature; SURF (Bay et al., 2006)), to binary string encoding with varying local sampling methods (oriented FAST rotated BRIEF; ORB (Rublee et al., 2011)), to binary scale- and rotation-invariant methods (binary robust invariant scalable keypoints; BRISK) (Leutenegger et al., 2011), to features that operate in the nonlinear scale space (compared to SIFT/SURF feature finding in the Gaussian scale space) (Accelerated-KAZE^9^(AKAZE) (Alcantarilla and Solutions, 2011). Table 1 summarizes the metrics and results from that analysis.

**TABLE 1:** Comparison of persistence and accuracy of state-of-the-art feature trackers on images of a principal-axis rotating small body at a body-image span of 150 pixels and a delta rotation of 1.25° per frame.

Algorithm	Average # of features/frame	Average track length	Track <19°	Track >44°	Error <2 pixels
AKAZE	31.4	20.6	32%	8%	62%
BRISK	40.0	18.2	44%	4%	92%
KLT	22.0	**38.8**	**13%**	**49%**	54%
ORB	41.5	16.3	51%	2%	86%
SIFT	**43.1**	16.4	48%	1%	**97**%
SURF	13.9	17.2	51%	4%	74%

“Track” refers to the number of frames tracked for a given feature. Error is the maximum error over a track.

For each algorithm, we used the assumption of a principal-axis rotator, the estimate of the spin rate, and constraints to avoid features on the body boundary. Such prior information is used to filter outliers. The analysis showed that there is a trade-off between the duration that a feature is tracked for and the accuracy with which it is tracked. SIFT, for example, has many features that are mostly accurate, but hardly any features that last for more than 44° of body rotation. In contrast, the KLT optical-flow method has many features with long tracks, but these features tend to drift with only a few low-error features. Feature descriptors, such as SURF and BRISK, could handle gradient variations and lighting changes, but they tend to fail with sharp changes in the shadow-dominant appearance from surface topography and boulders. BRISK has slightly longer average feature tracks than SIFT but has more erroneous features. A key finding here is that none of these algorithms adequately met all the required characteristics. The reason for this is primarily due to the change in lighting as the body rotates. See the study by Morrell (2020) for more details.

Key findings from the analysis of the feature-tracking algorithm for small bodies include the following:

1) Current methods cannot provide ideal characteristics for feature tracking; hence, the choice of method should consider the downstream use (i.e., how they will be used in orbit determination)
2) For improved performance, with the changing lighting, each component of feature tracking needs to be considered:
  a) Extraction (localization) of features
  b) Matching of features, including feature description
  c) Outlier rejection for sets of matched features
  d) Loop closure to recognize when features are re-observed

Some work has been done to learn descriptors for this domain to improve robustness to lighting and perspective changes. A convolutional neural network (CNN) was trained to match image patches through the lighting and perspective changes of a rotating body using a set of randomly generated bodies (see Section 5.2). After training, the network was evaluated on the real imagery of comet 67P. The feature-matching performance outperformed both SURF and BRIEF (Calonder et al., 2010) after approximately 400 training epochs (see Harvard et al. (2020), Villa et al. (2020) for more details). While these results are quite promising, further development to couple matching with feature extraction is needed to evaluate the overall performance, as feature extraction remains susceptible to tracking moving shadows.

Ongoing work on classical feature descriptors includes the use of adaptable filter parameters for the rotation rate and the body-image span and then comparing them to neural network–based equivalents. In our Experiment Section (Section 5.3), we use a subset of state-of-the-art feature trackers, with acknowledgement of the trade-offs that are required in doing so. Specifically, we evaluate the long feature tracks of optical flow with their larger errors and the short tracks of SIFT with their smaller errors.

### 4.9 Establishing a Coarse Shape

After tracking features for one full rotation, the orbit determination algorithm generates a set of post-fit 3D landmarks (Section 4.3). These landmarks and the pole-from-silhouette (Pfs)-generated visual hull are then used to generate a true coarse shape. The goal of this watertight mesh shape is to produce an initial gravity model and seed the dense 3D reconstruction, which is later used to identify candidate landing sites. As the spacecraft approaches the body, the number of landmarks gradually increases, from a few up to thousands. The key challenges for coarse mapping are handling sparse features at the start of this phase, managing holes due to missing information, and dealing with large shadowed regions of the body, where few landmarks, if any, are detected.

**FIGURE 4:**
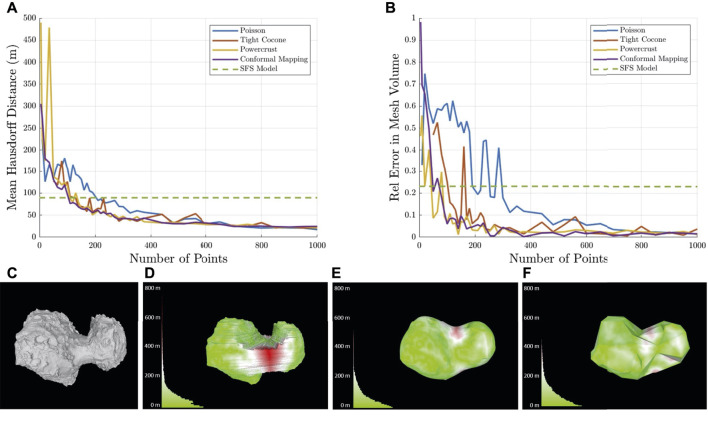
Comparison of 3D coarse mapping algorithms with varying numbers of landmarks. **(A)** Mean Hausdorff distance. **(B)** Error in volume relative to the ground truth. Example of coarse reconstruction of comet 67P with 100 landmarks with colored histograms of distance to the ground truth. **(C)** Ground truth, **(D)** shape-from-silhouette model, **(E)** Poisson surface reconstruction model, and **(F)** spherical conformal mapping model.

To handle the aforementioned challenges, we investigated leading approaches for generating meshes from landmarks. The selected algorithms can all generate watertight meshes even with regions devoid of landmarks. We focused on the impact of the number and distribution of landmarks on the quality of the generated meshes. Table 2 summarizes the characteristics of the selected algorithms, and Figure 4 shows their corresponding results.

**TABLE 2:** Summary of coarse 3D mapping methods.

Algorithm	Desired inputs			Desired outputs		
	Can use sparse features	Amenable to incremental data	Does not require normals	Interpolates	Generates isometric mesh	Generates watertight shape
Screened Poisson surface reconstruction Kazhdan and Hoppe, (2013)	N	N	N	Y	Y	Y
Powercrust Amenta et al. (2001)	N	N	Y	Y	N	Y
Tight Cocone Dey and Goswami, (2003)	N	N	Y	N	Y	Y
Spherical conformal mapping Choi et al. (2016)	N	N	Y	N	Y	Y
Visual hull deformation	Y	Y	Y	Y	Y	Y

Screened Poisson surface reconstruction is a widely used algorithm that requires the surface normal of landmarks to form a continuous vector field to generate an isosurface. The vector field is an approximation of the gradient of the indicator function, a binary function that identifies points inside the body, which is derived by solving a Laplace equation. Powercrust and Tight Cocone both rely on the construction of the 3D Voronoi diagram and use the Voronoi vertices to approximate the landmark normals. Powercrust uses the intersection of the internal and external maximal balls, located on the approximated medial axis, to define the surface. Alternatively, Tight Cocone uses the principle of co-cones to construct the Delaunay triangulation and then peels the convex hull to fill holes and enforce a watertight mesh. Spherical conformal mapping introduces an additional constraint, enforcing a genus-0 reconstruction, an assumption which is consistent with known small bodies (i.e., no doughnut shapes or ones with open interior holes). The algorithm enforces this by first mapping the 3D features to the surface of a sphere. The Delaunay triangulation is then constructed to determine feature connectivity, which is applied to the original model to construct the surface.

Figure 4 compares the performance of these algorithms using comet 67P for differing numbers of landmarks. All these algorithms converge toward a similar result with a higher number of landmarks. However, with a smaller number of landmarks, the Poisson and Powercrust methods perform poorly due to interpolation of new vertices that are not consistent with the ground truth. An example of the results produced by each algorithm at 100 points is shown in Figure 4.

While we have shown that watertight shapes can be constructed to a reasonable degree of accuracy with a large number of landmarks, an algorithm that can incrementally, continually, and efficiently refine the shape of the already watertight visual hull, starting with sparse landmarks and extending to dense landmarks, would be ideal because it would incorporate all prior measurements. A method to continually and efficiently deform the visual hull is being investigated, starting with few landmarks. The visual hull can also be used as an outer bound on the mesh generation process to reject outlier landmarks and limit interpolation (such as in Poisson reconstruction). The most suitable implementation for our estimation pipeline for coarse mapping is still under investigation as the choice will depend on accuracy metrics for subsequent dense matching and the ability to efficiently incorporate new landmarks.

### 4.10 Refining the Shape for Selecting Viable Landing Sites

While a coarse model is sufficient for establishing an initial gravity model for closer approach, a finer shape model is needed for improved gravity and surface models to identify an initial set of candidate landing sites. Such a model requires a denser 3D reconstruction of the body shape. To produce this refined shape, we generate dense depth information for all pixels in a set of reference images and fuse them into a 3D mesh. Depth information is generated using a multi-view stereo approach, where corresponding pixels are matched over multiple images, and the body-relative motion of the camera among the images is used to triangulate the depth. A critical prerequisite to dense mapping is an accurate estimate of the spacecraft’s trajectory relative to the body. This is only possible after numerous, highly reliable, and evenly distributed features have been tracked during close approach.

Out of the numerous approaches to multi-view stereo, we implemented a pixel-wise Bayesian depth-estimation approach (called REMODE (Pizzoli et al., 2014)), with the ability to seed the estimation with initial guesses from the 3D to the 2D projection of the coarse 3D model. Depth is estimated for a set of reference frames (e.g., every 15° of body rotation) and is projected out to 3D points using the camera model. The set of these 3D point clouds can be used to further refine the relative spacecraft trajectory through alignment of the overlapping parts of the point cloud using an iterative closest point method (Besl and McKay, 1992). The set of point clouds can then be merged into a combined 3D representation using a signed-distance field with the Voxblox algorithm (Oleynikova et al., 2017). From the signed-distance field, the fast-marching-cubes algorithm (Lorensen and Cline, 1987) is then used to extract a mesh, with further post-processing required to make the mesh watertight.

**FIGURE 5:**
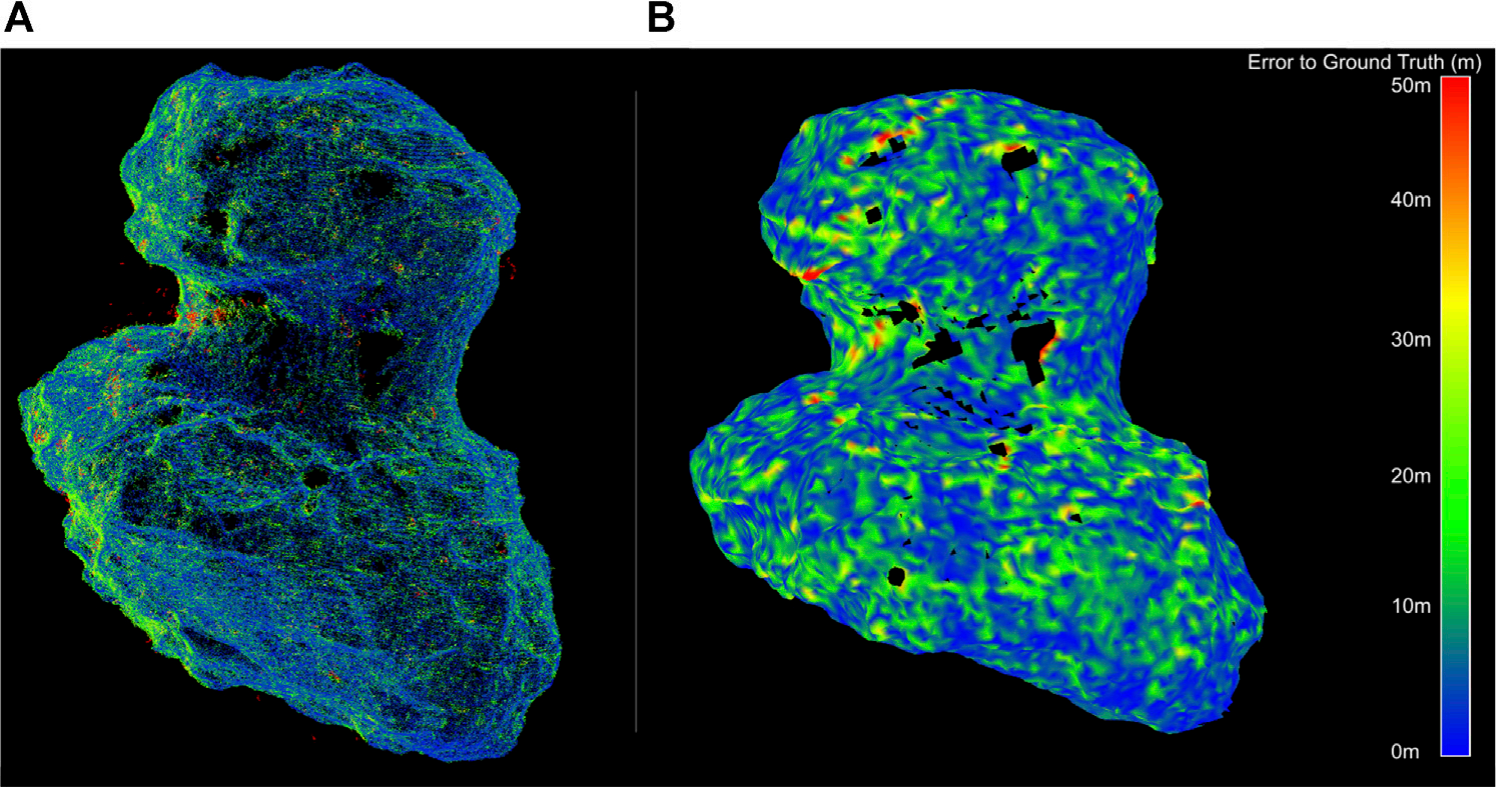
Example performance of dense 3D mapping algorithms on comet 67P at a body-image span of 1000 pixels (at a distance of 50 km for a 4.3 km body diameter) at a delta rotation of 0.25° per frame (i.e., a 30-s imaging interval). **(A)** Down-sampled accumulation of point clouds from depth reconstruction using REMODE (Pizzoli et al., 2014). **(B)** Filtering of the point clouds into a signed-distance field from which a mesh is extracted (using Voxblox (Oleynikova et al., 2017)).

The dense mapping approach can be applied on a global scale, to provide a higher-resolution shape to select candidate landing sites, and at a higher resolution on a local scale, to assess geometric hazards at candidate sites. While these approaches generated promising results with accurate knowledge of the trajectory (see Figure 5), the approaches are sensitive to the accuracy of the relative trajectory, placing requirements on the maximum uncertainty levels permissible before entering this phase.

### 4.11 Identifying Candidate Landing Sites

Perhaps the most scrutinized and manually controlled event in past small-body missions has been the touch-down or landing site selection. In current missions, this process can take many months and thousands of hours. Fundamentally, the surface environment of small bodies presents, by far, the highest level of uncertainty and risk to the spacecraft. Since the topography and surface properties of small-body surfaces can vary wildly, great care should be taken to evaluate all potential risks to the spacecraft before landing is attempted.

To identify candidate landing sites, we have to consider all potential hazards associated with the surface environment, including stability of the spacecraft on the surface, the thermal environment, potential for dust contamination, entrapment, and poor visibility of the Sun (for power) or Earth (for communication). Some risks may be mitigated through careful design of the spacecraft (see Section 5.1). Others depend on the shape of the body, the global orientation of the landing site, and the local surface properties. Here, we focus on three key potential hazards that the spacecraft will have to reason about: the surface stability, thermal environment, and line-of-sight constraints, all of which require a detailed 3D map on the scale of the spacecraft. Both map resolution and uncertainty of the map can be improved using both narrow- and wide-FOV cameras by maneuvering the spacecraft in proximity of the body prior to landing.

• Surface stability: We use the coarse-shape and gravity models to exclude steep-sloped regions where the platform would not be stable on the irregular gravity-field body, thus focusing subsequent high-resolution observations on largely flat or low-sloped regions. The spacecraft design and concept of operations drive the slope-stability requirement (see Section 5.1 for details).
• Thermal environment: Characterizing thermal hazards autonomously from a SmallSat is challenging because it requires either a large thermal-IR imager or computationally intensive thermal–physical models. However, if the spacecraft has a directional radiator to reject heat by facing the cold sky, one can consider a conservative approach to identify and avoid highly concave (“hot traps”) areas on the surface, where a view of the sky may be occluded (i.e., ambient occlusion).
• Line-of-sight occlusions: Similarly, the line of sight of the Sun is important for power and illumination for imaging and that of the Earth is important for communication, at least, for a portion of the day. We use the pole orientation and the shape model to compute the diurnal visibility maps of the surface for known Sun and Earth positions. Autonomous spacecraft may be designed to be tolerant of short-duration occlusions for science investigations.

**FIGURE 6:**
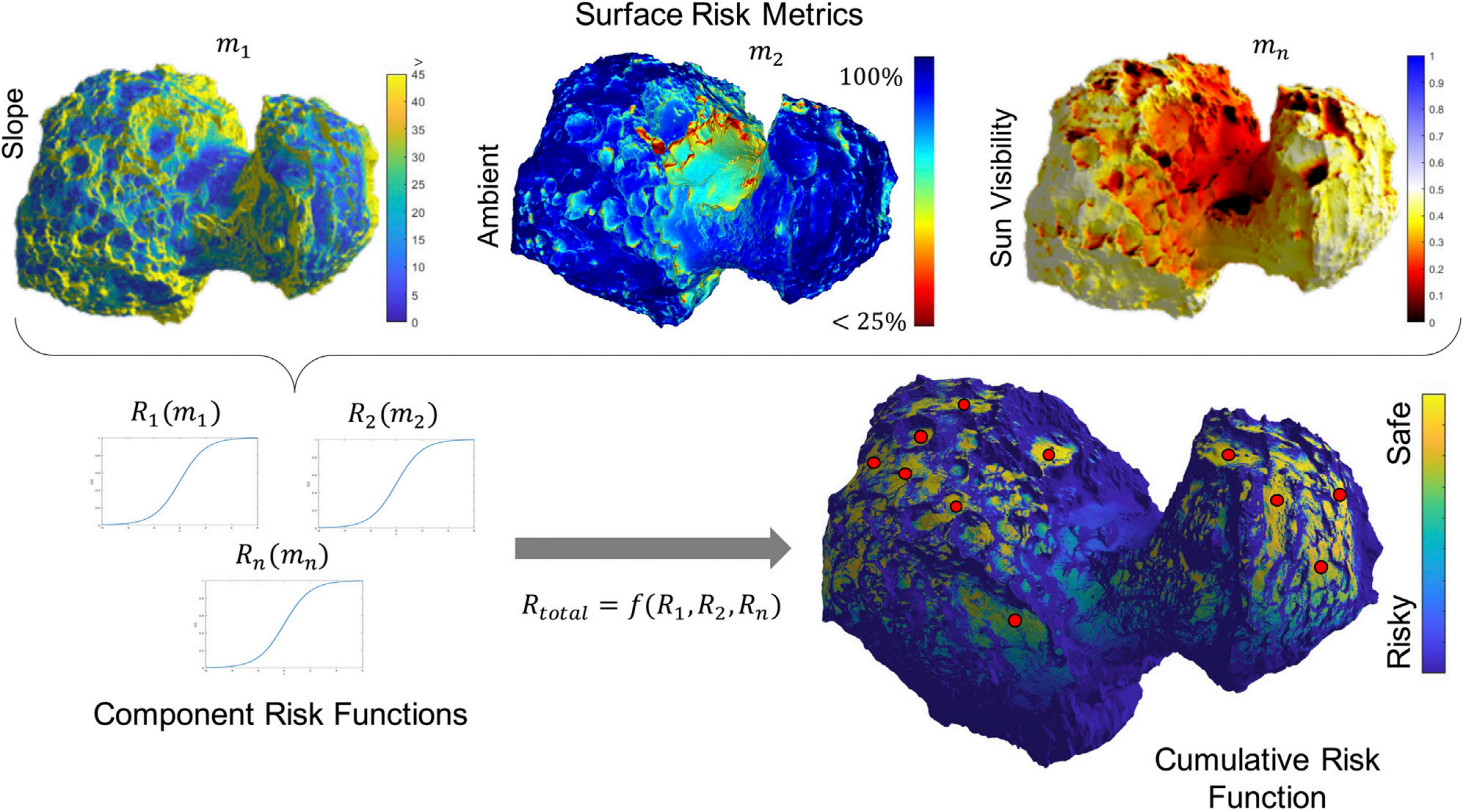
Illustration of the landing-site hazard assessment and selection process. **(A)** Spatially distributed risk metrics computed for a coarse global model, with local refinement in promising regions. **(B)** Convolution of multiple risk metrics into a cumulative risk metric to identify safe candidate landing sites.

Figure 6 illustrates the risks associated with the surface stability, thermal environment, and line-of-sight solar occlusions for comet 67P assuming zero obliquity (i.e., the Sun is at 0° latitude). Our initial conservative strategy is to convolve these cross-discipline risks to identify candidate landing sites, imposing allowable thresholds on each independent metric. Ongoing research is investigating a more sophisticated strategy for decision-making and actions.

## 5 The Experiment

### 5.1 Notional Spacecraft Architecture

**FIGURE 7:**
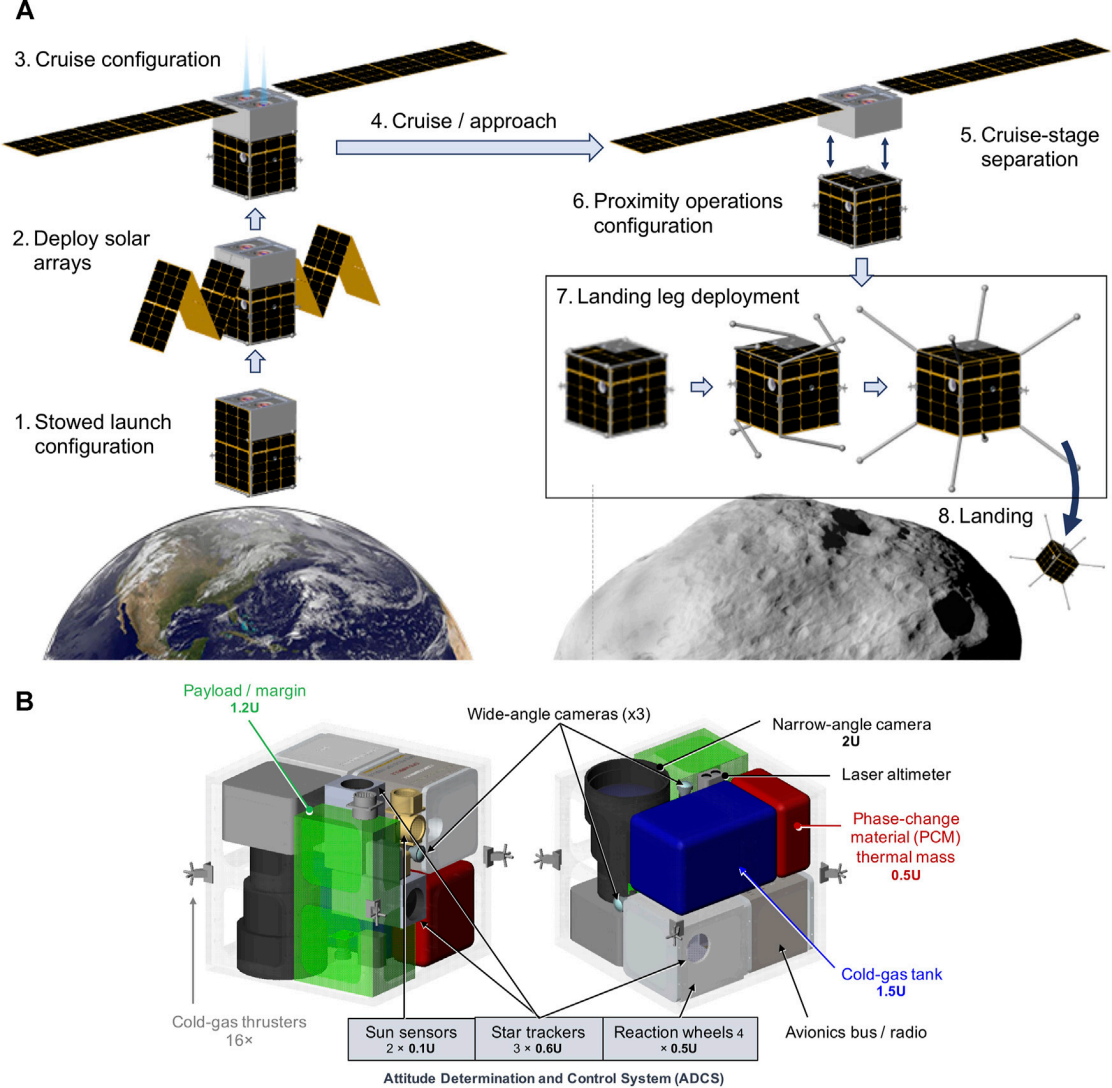
**(A)** Spacecraft architecture for approach and landing on a small body. **(B)** Internal configuration of notional spacecraft architecture with key components (or volume allocations). The cubic chassis is protected by eight corner-mounted “legs” for landing on any side.

Safe landing depends on the body/surface characteristics and on the spacecraft capabilities and vulnerabilities. To address the latter, we developed a conceptual spacecraft design based on existing (or near-term projected) SmallSat technologies. This design allows us to consider realistic guidance, navigation, and control (GNC) constraints when developing our autonomous approach, landing, and mobility. The design, shown in Figure 7, leverages a study of the accessibility of NEOs using SmallSats (Papais et al., 2020). A key technology for a SmallSat to rendezvous with an NEO is propulsion. With a low-thrust Δ*V* of about 3 km/s, a small population of 100 NEOs larger than 30 m in diameter could become accessible using solar electric propulsion (SEP). SEP would require deployable solar arrays, which would later interfere with landing and mobility on the surface. As a result, we designed the spacecraft to jettison the SEP engines/solar panels prior to descent. For repeated landings and robust surface mobility, we chose spring-loaded omnidirectional landing legs, which allow the spacecraft to touch down in any orientation (Hockman et al., 2017). To relax requirements on resolving slopes on the scale of the spacecraft, our self-righting spacecraft design would be able to tolerate tumbling down short slopes on the order of tens of meters.

### 5.2 High-Fidelity Mission-Data Generation

**FIGURE 8:**
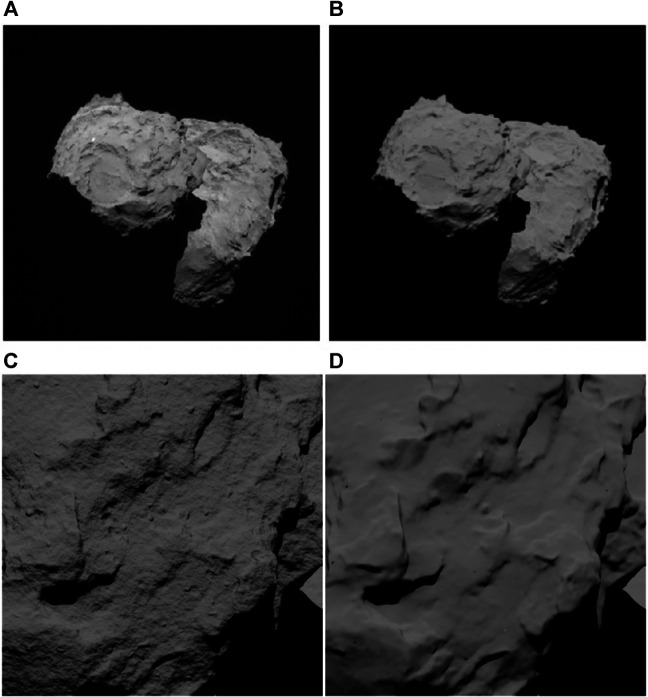
Comparison between real **(A)** and synthetic **(B)** images of comet 67P using the Hapke model. The real image is from Rosetta’s NAVCAM. Synthetic image of comet 67P with **(C)** and without **(D)** procedural terrain generation. Bump mapping is used, in combination with multiple terrain-noise functions. The Hapke model is used for reflectance.

Our autonomous approach and landing requires imaging more frequent by an order of magnitude than what is typically available from past missions. Frequent imaging is possible for onboard autonomy because the spacecraft is not constrained by communication and ground operations. For development and testing, we used real images and telemetry from prior missions (e.g., Rosetta’s images of comet 67P) and images synthetically generated using high-fidelity models of lighting, optics, and trajectories. Based on the open-source, physics-based rendering engine provided by Blender Cycles, we developed a framework for generating and rendering high-fidelity, photorealistic images of small bodies. The framework is capable of 1) generating realistic images of terrains across vast scale changes throughout the approach phase, 2) accounting for multiple light reflections and scattering, and 3) supporting custom reflectance properties of regolith-covered surfaces (Figure 8).

#### 5.2.1 Procedural Terrain Generation

When working across large-scale changes (approaching a kilometer-sized body from millions of kilometers down to the surface), the 3D mesh of the target body has to support a vast range of imaging resolutions. To handle such scales, we use a technique known as procedural terrain generation, which uses multi-octave noise functions to perturb the surface within a single facet of the mesh when the camera gets progressively closer to the body. Such functions can produce multi-octave random noise and patterns that resemble boulders, craters, and other natural features typical of small bodies. The algorithmic terrain can be generated in two ways: either *via* direct displacement of the facets in the 3D mesh or by bump mapping, a technique which only modifies the surface texture to resemble 3D displacements. The latter is computationally more efficient and produces results that are sufficiently accurate that they can be used during the feature-tracking phase. Figure 8C,D show a comparison of two images rendered with and without procedural terrain generation from a fixed-resolution mesh of comet 67P using bump mapping.

#### 5.2.2 Multiple Reflections and Light Scattering

To render photorealistic images, we use state-of-the-art ray-tracing techniques using Blender Cycles software. The software traces non-collimated rays from a light source, which emulate the natural paths of photons (e.g., originating from the Sun), accounting for multiple reflections and scattering and potentially modeling refraction through transparent media (e.g., water or glass). Finally, the rays reach the observer (i.e., the camera), where the quantity, distribution, and orientation of rays render the scene to a high fidelity. When the body occupies a sub-pixel in the image, we render the body in the image using a synthetic point-spread function (PSF). As the body grows beyond a few pixels, we switch to using ray tracing from approach through landing. Far bodies and background stars are also rendered using a PSF. To generate the full scene, we merge the rendered small-body foreground with a separately rendered star-field background.

#### 5.2.3 Regolith Reflectance Models

The unconventional optical properties of regolith-covered terrains, such as the Moon, asteroids, and comets, challenge standard reflectance models, such as the Lambertian and Oren–Nayar models, which fail to capture their distinctive behaviors. One dominant effect is the shadow-hiding opposition effect, which manifests as a surge in brightness when the sun-phase angle is close to zero with respect to the observer. We developed custom models in Blender using its open shading language (OSL) interface. The first is the Hapke model (Hapke, 2012), a state-of-the-art reflectance model tailored to the optical properties of regolith bodies. The second is the commonly used Lommel–Seeliger model (Fairbairn, 2005).

#### 5.2.4 Camera Model, Calibration, and Validation

Our models include the camera coordinate transformation relative to the spacecraft, lens model (field of view, distortion, aperture, depth of field, and chromatic aberration), and camera model (sensor type (RGB vs. gray scale), image resolution, bit depth, pixel pitch, exposure time, motion blur, read noise, dark current, and dynamic range (up to 20 stops)) using Filmic Blender. When using ray tracing, image exposure is modeled as a pixel-intensity scale factor and should be calibrated using a real camera system to properly represent the actual photon integration time. Figure 8 compares an image rendered using the Hapke reflectance model and the actual image from the Rosetta mission (some camera parameters of the Rosetta mission are not available and a constant, average albedo is used for the surface in the synthetic image).

### 5.3 Mission Simulation: Results and Discussion

To test our estimation pipeline for the autonomous approach, we designed mission simulation experiments to mimic realistic approach scenarios. One group from our team was responsible for generating the artificial bodies using the high-fidelity simulation described in Section 5.2 and telemetry^10^ based on the notional spacecraft described in Section 5.1. A second group was responsible for executing the algorithms of the various phases of the pipeline, seeding estimates and uncertainties from one phase to the next and executing orbit determination across all phases, as shown in Figure 1. The manual execution of the individual algorithms was necessary because our framework consists of several tools and algorithms that are not yet integrated into a single software implementation. In each approach scenario, we used a preplanned spacecraft trajectory and produced trajectory corrections based on estimates and uncertainties that were computed at the different phases. A total of two experiments were completed, with the first primarily used to debug the pipeline. Below are the results from the second experiment, where 14 image sets were generated, each with hundreds of images, as the spacecraft approached the target from thousands of kilometers away down to 20 km away. The results show the recovery of the body-relative trajectory, rotation, and shape and the comparison to the ground truth.

**FIGURE 9:**
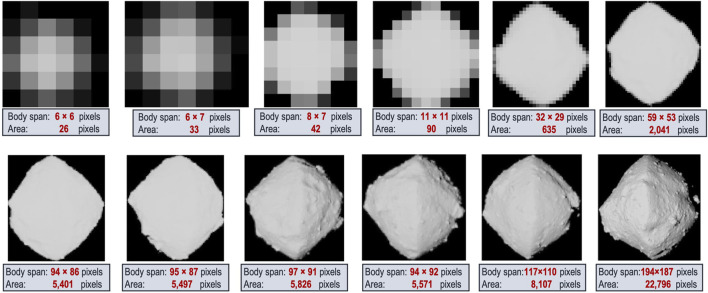
First image in image sets 1–12 of the experiment.

**FIGURE 10:**
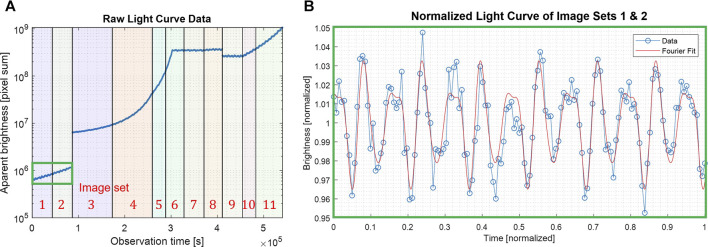
Periodicity analysis of the experiment. **(A)** Raw light curve showing general increase in brightness during approach and discontinuities associated with maneuvers and changing exposure settings. **(B)** Periodicity analysis of the light curve from image sets 1 and 2 only. After normalization, a 4th order Fourier fit estimates the period to be *P* = 3.728 ± 0.006 h.

Figure 9 shows the region of interest in select images acquired using the narrow-FOV camera during the approach (for clarity, we adjust the display size of the pixel in these images). Once the body is detected in the navigation camera, one of the first quantities to be estimated is the body’s rotational period, as discussed in Section 4.5. In practice, this would begin when the body is sub-pixel in size, more than a million kilometers away. However, we started our experiment at 3,000 km, where the body-image span was six pixels across, primarily focusing on validating spatially resolved bodies. Figure 10 shows the result of the periodicity analysis for the first two image sets, with a period estimate of *P* = 3.728 ± 0.006 h. This initial data set covers approximately six full rotations and has a very coarse imaging frequency every 10 min. Thus, the period is only estimated with a relative accuracy of 10^–3^. With more frequent imaging and extensive integration, the light-curve data would reduce this uncertainty by several orders of magnitude.

**FIGURE 11:**
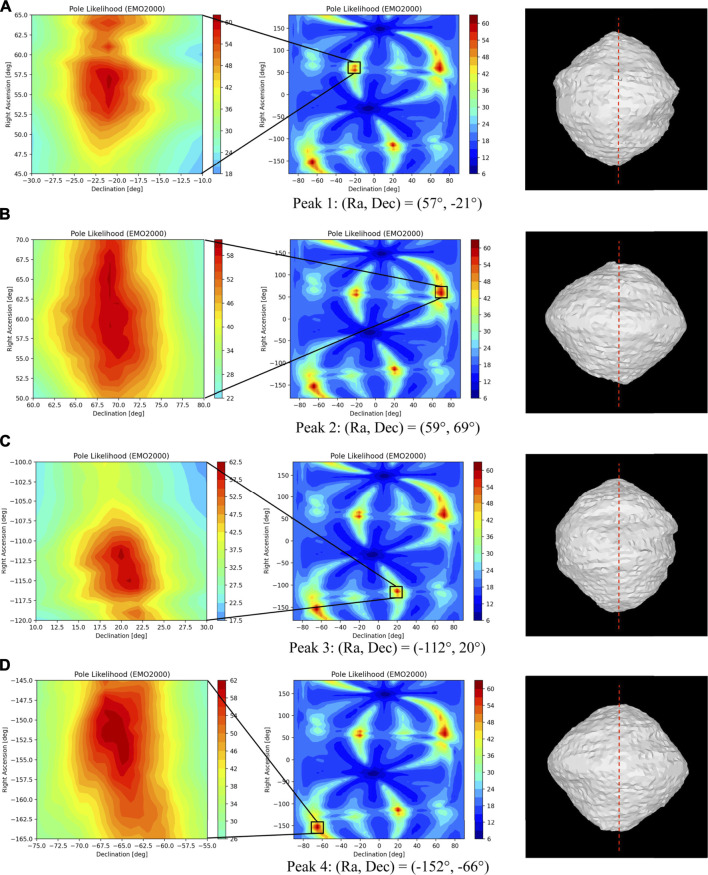
Pole-from-silhouette (PfS)/shape-from-silhouette (SfS) pole and shape estimation using image set 7.

Initially, when the body-image span was less than ten pixels, PfS and SfS were unable to resolve the pole and the visual hull. However, as the body-image span grew over thirty pixels, PfS and SfS were able to estimate the possible poles and generate the visual hulls. Figure 11 shows an example of pole estimation that generates four pole hypotheses and their corresponding visual hulls, which results from the observation symmetries described in Section 4.6. This ambiguity would be resolved in the subsequent feature-tracking phase.

**FIGURE 12:**
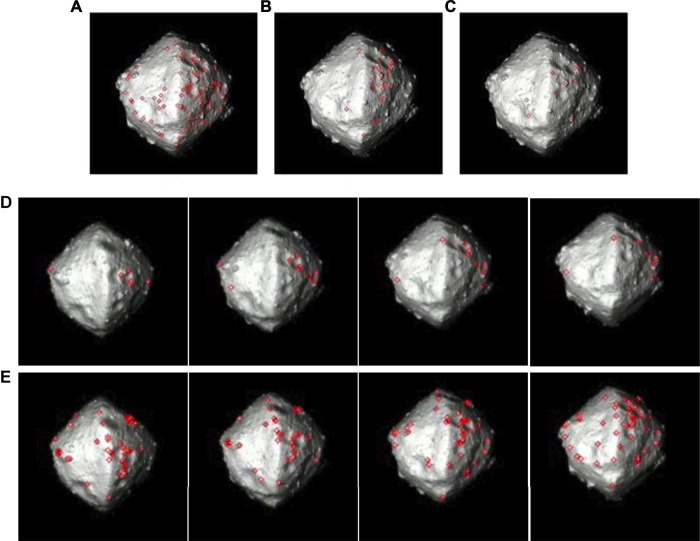
Example feature distribution for the different algorithms. **(A)** Optical flow, **(B)** BRISK, and **(C)** SIFT. **(D)** KLT tracking on sequential images with 10-min imaging. **(E)** KLT tracking on sequential images with 2.5-min imaging. All other parameters are unchanged.

The analysis of feature tracking in this blind experiment affirms the aforementioned results of Section 4.8. In general, optical-flow approaches are able to track features at farther distances (i.e., smaller body-image span), track more features, and track features for longer durations (i.e., larger rotational angles), when compared to feature-matching approaches, such as SIFT and BRISK. That is why optical flow was selected for the earlier stages of the approach. However, once more details become visible in the image of the body (typically, when the body-image span >150), SIFT and BRISK are able to identify more features and appear to have lower error. After this point, the trades continue between choosing longer tracks prone to drift and shorter tracks with less drift (see Figures 12A–C for a comparison of the number and distribution of features). Another finding is the high sensitivity of these feature-tracking algorithms to the imaging rate. It turns out that there is a substantial impact on the tracking performance of all algorithms when the imaging period was decreased from 10 to 2.5 min, as shown in Figures 12D,E. In an autonomous mission, the imaging rate should be set to limit the rotations per frame in order to have sufficient similarity and overlap between consecutive images, which is characterized by a pixels/frame velocity of feature movement. For this particular data set, the maximum acceptable velocity was estimated to be 7 pixels/frame (with a search window of 25 × 25 pixels across four pyramid levels). The best results were obtained by tuning the algorithms to track many features and then post-filtering them based on the expected bounds of feature movement, as dictated by the body-image span and the rotation rate.

**FIGURE 13:**
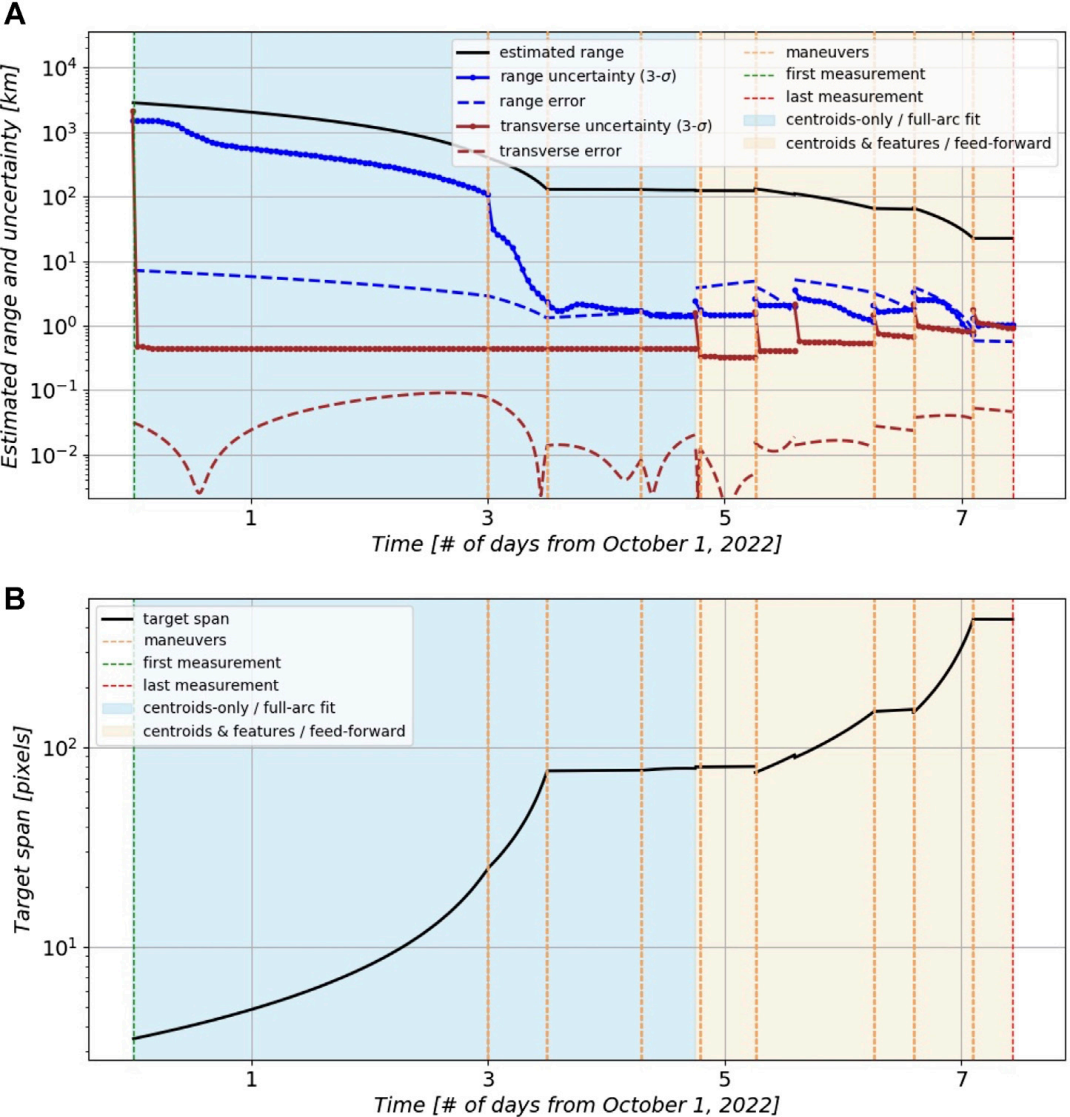
**(A)** Range and transverse estimates, uncertainties, and errors relative to the ground truth across the entire approach from 3,000 km down to 20 km (image sets 1–14). **(B)** Estimated body-image span across the same approach.

Orbit determination uses the information generated by the above algorithms to estimate the trajectory of the spacecraft relative to the unexplored small body and estimate its rotation, such as the spin rate and the pole, and its associated landmarks. After the experiment was complete and the ground truth was revealed to the estimation group, we compared our estimates during the approach to the ground truth data. Figure 13 shows the range and transverse estimates, uncertainties, and errors as well as the body-image span during approach for image sets 1 through 14. As expected from optical measurements, throughout the entire approach, range errors were at least an order of magnitude larger than transverse errors. Periodic updates from the orbit determination keeps the target within the camera field of view. Based on estimates at different phases, eight trajectory correction maneuvers were executed to get closer to the target and control the approach latitude.

During approach, the estimation framework maintained a range uncertainty that was an order of magnitude smaller than range estimates. Thetransverse error remained below 100 m, which is ideal to ensure that the target remains in the camera’s FOV for extended periods. Transverse errors are routinely smaller than their uncertainty metrics. In the first phase of the approach, only body-centroid measurements were used for orbit determination (image sets 1–8; the blue-shaded area in Figure 13). Estimates in that phase used data arcs that included all prior centroid measurements. However, when tracked features were added (image set 9 onward) to the estimation process, a feed-forward approach, which segments the data arcs into smaller sets, was necessary to manage the growth in data from the accumulation of landmarks tracks. In the feed-forward approach, we use estimates and uncertainties from the prior segment. To prevent divergence, at the start of each feed-forward segment, the prior covariance diagonals are amplified (here, by a factor of two) and then fed into the current segment. This covariance inflation process is similar to process noise that is used in a Kalman filter, where prior information is de-weighted to account for system-modeling errors. For image sets 9–12 (days 4.8–6.5), the range errors exceeded range uncertainties but remained within the same order of magnitude. This may have resulted from optimistic uncertainties that were fed-forward from the full-arc solution of image sets 1–8. The periodic inflation of the covariance in the feed-forward approach provides robustness in such situations, as evidenced by the bounded uncertainties in the last two image sets (13–14). To mitigate issues associated with this error–uncertainty discrepancy in image sets 9–12, it may be useful to utilize the feed-forward mechanism during the earlier portions of approach (i.e., image sets 1–8). The large uncertainties, especially those of the transverse, may have resulted from the higher weighting of the centroids relative to the landmarks. This is poignant in image sets 13–14 (the last two segments), where transverse uncertainty seems to grow and become comparable to range uncertainty. Uncertainty in the centroid grows with the increase in the body-image span. Adjusting the weighting of the different sources of measurements is the subject of ongoing research.

**FIGURE 14:**
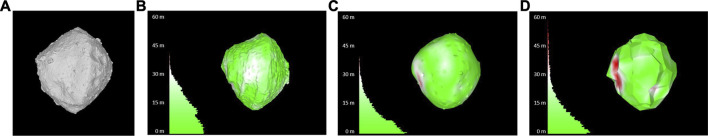
Example coarse mapping results from the experiment, using 300 features. **(A)** Ground truth, **(B)** shape-from-silhouette model, **(C)** Poisson surface reconstruction model, and **(D)** Powercrust model.

When feature tracking performs consistently and accurately, the output of the 3D landmarks was able to generate a coarse 3D model. Results from the 3D shape reconstruction were obtained using three hundred landmarks. When fewer landmarks are available, and depending on the concavities of the body, the visual hull would produce the lowest errors. However, as the number of features grows to the hundreds, the 3D shape reconstruction algorithms would outperform the visual hull as they would generate a true shape of the body. Figure 14 and Table 3 compare the performance of several reconstruction algorithms to that of the visual hull. Ongoing work is investigating algorithms that can work iteratively as the number of landmarks increases during approach, starting with the visual hull information.

**TABLE 3:** Summary of coarse 3D mapping results with 300 features.

Algorithm	Mean Hausdorff distance (m)	Maximum Hausdorff distance (m)	Volume error	Center-of-volume error (m)
Shape-from-silhouette model	10.6	38.5	6.54%	6.40
Screened Poisson surface reconstruction	8.16	39.5	0.41%	5.84
Powercrust	9.33	56.1	5.47%	8.38
Cocone	8.61	59.9	7.85%	6.36

When compared to the ground truth, estimates and uncertainties of body rotation parameters showed promising results for establishing situational awareness for a principal-axis rotator (Table 4). The pole RA and Dec angles are expressed in the Earth mean orbital (EMO) frame, to avoid the singularity in the Earth mean equatorial (EME) frame. Note that estimates of the pole’s RA and Dec were fairly accurate, but the uncertainties were too optimistic. Future work will focus on generating more realistic orbit and pole uncertainties in the orbit determination framework.

**TABLE 4:** Comparing estimated body rotation parameters to the ground truth.

Parameter	Units	Ground truth value	Estimated value and 1*σ* uncertainty	References frame
Spin rate	°/day	2319.46309	2319.464 ± 0.010	—
Spin period	hours	3.725	3.724999 ± 0.000016	—
Pole’s RA	°	0.0	—	EME Frame
	°	90.0	90.38 ± 0.11	EMO Frame
Pole’s Dec	°	90.0	—	EME Frame
	°	66.56	66.280 ± 0.040	EMO Frame

This work tackles a key challenge toward the autonomous approach and landing on small bodies, but much work remains to achieve such a goal in a flight mission. The algorithms presented herein require further development, refinement, and characterization. Once matured, the algorithms’ computation load (computational cycles and memory) would have to be assessed. In the scenario described in this article, the spacecraft response can be slower because of the slow dynamics of proximity operations around small bodies. The guidance, navigation, and control functions require integration with propulsion, thermal, power, and communication subsystems and with system-level autonomy algorithms. Such algorithms manage resources, plan activities, assess system health, and execute actions. Progress on these has been made under separate efforts, but they require further development and maturation (Chien et al., 2004; Fesq et al., 2019). Recent work that investigated closing the guidance loop using meta-reinforcement learning in a simplified lunar landing scenario pointed out the potential of applying machine learning not only on the estimation side but, potentially, on mapping beliefs to actions (Furfaro et al., 2020).

## 6 Conclusion

We have presented a multi-phase estimation framework for a notional spacecraft design that would be capable of autonomously approaching, rendezvousing with, landing, and moving on small unexplored bodies, whose physical properties (spin rate, pole, shape, surface, mass distribution, and surface topography) may be unknown or poorly constrained *a priori*. Results from a blind experiment that mimicked a realistic mission scenario showed an autonomous approach from 3,000 km down to 20 km, with a total of eight trajectory correction maneuvers, while keeping the body within the narrow-FOV camera *via* periodic orbit determination updates to the estimated trajectory. At the start of the approach, the body centroid was used to adjust the relative trajectory. Then, light curves were used to estimate the body’s spin rate to an accuracy of 22 s or 0.16*%* of its 3.74 h period. That estimate, together with the centroid-updated relative ephemeris, was used to estimate the pole from the silhouette starting at a body-image span of thirty pixels. Using shape-from-silhouette, four poles and corresponding shapes formed viable hypotheses. The next phase used optical flow to track features over a long duration, reaching body-rotation angles of 120°–160°. The pole hypotheses, shape-from-silhouette, and feature tracks were then used in the orbit determination to refine estimates, compute uncertainties, and disambiguate hypotheses. The estimated relative trajectory during approach maintained a 3*σ* uncertainty of more than one order of magnitude below the estimated range, keeping the spacecraft safe from the body. Post-fit 3D landmarks were used to generate a true coarse shape of the body with a mean Hausdorff error of <11 m or 2*%* of the 480 m diameter body.

This blind experiment demonstrated the viability of an autonomous estimation framework to establish knowledge of a principal-axis rotating body of unknown motion and rotation parameters. Much work remains to be done to substantially mature these capabilities, but we consider this step as a promising start. Ongoing research is focusing on conducting blind experiments for getting from 20 km down to the surface, which includes dense 3D reconstruction and hazard assessment for landing based on algorithms described here. It also includes maturing and validating the framework on a wider range of bodies to further characterize the evolution of uncertainty during approach and landing.

Today, small-body missions heavily rely on lengthy ground-in-the-loop operations that constrain the spacecraft maneuvers because they rely on stale downlinked data due to communication delays and time for operations planning. These preliminary results show promise for the ability to conduct such missions autonomously in the future. Moreover, it would enable greater and more affordable access to multiple diverse targets and multi-spacecraft access to a single target, offering flexibility and robustness given redundant assets. The development of the framework and simulation tools enable the continued development, maturation, and testing of autonomous capabilities under realistic conditions.

Developing autonomous spacecraft to reach and explore the surfaces of small bodies not only expands access to bodies of interest to science, planetary defense, *in situ* resource utilization, and possible human exploration but also provides an opportunity to advance space autonomy. For near-Earth objects, this can be done using SmallSats technologies to mature autonomy in perception-, estimation-, decision-making–, and action-rich scenarios. Near-Earth objects embody many challenges of more remote destinations. However, the slow dynamics of interacting with such low-gravity bodies affords time for redundant onboard sensing (repeated imaging and multiple cameras), processing, managing uncertainty, reconciling conflicted estimates, building *in situ* models, deciding, and taking actions. All this will occur while operating in adequately challenging environments from a perception and decision-making standpoint but with more benign physical interaction and impact. After all, moving and falling onto the surface of a microgravity body would be much gentler on the spacecraft than moving and falling onto the surface of larger bodies. As such, we consider the development of autonomy for such targets to be a key stepping stone toward exploring more complex and challenging destinations.

## Data Availability

The raw data supporting the conclusion of this article will be made available by the authors, without undue reservation.

## Footnotes

1 Mission Concept Studies for the Planetary Science and Astrobiology Decadal Survey 2023-2032, https://www.hou.usra.edu/meetings/pmcs2020/

2 2018 Workshop on Autonomy for Future NASA Science Missions: Output and Results ‐ Small body Design Reference Mission https://science.nasa.gov/science-pink/s3fs-public/atoms/files/NASA%202018%20Autonomy%20Workshop%20Small%20Bodies%20DRM%20reports.pdf

3 The gold standard for state of the practice is to use Shape-Photoclinometry (SPC), but this requires human input to reconstruct a 3D shape from landmarks. In this work, we have evaluated photogrammetry-based techniques for the autonomous pipeline.

4 Autonomous in-space navigation was used on Deep Space I, Deep Impact, and Stardust.

5 The data arc is the collection of measurements used in a single batch estimate to produce a trajectory update.

6 The accuracy of the rotation rate estimate is on the order of 0.001°, the equivalent of a less than a second in a twenty-five-hour period.

7 A zero sun-phase angle is when the Sun is directly behind the spacecraft along the approach line toward the body.

8 The pole points both towards the Sun and the spacecraft approach vector.

9 KAZE means wind in Japanese. This word was used to symbolize the nonlinear processes that govern air flow on a large scale.

10 Telemetry includes initial navigation data of both the body and the spacecraft with typical large uncertainties before the start of the approach. It also includes realistic imagery of the unknown small-body during approach.
