# Supplementary material for: Autonomous Exploration of Small Bodies Toward Greater Autonomy for Deep Space Missions
Source: Front Robot AI. 2021 Nov 1;8:650885. doi: 10.3389/frobt.2021.650885 (PMC8592236; doi:10.3389/frobt.2021.650885)
Supplement: Supplementary file 1 [file DataSheet1.pdf]

Supplementary Material

1 SUPPLEMENTARY TABLES AND FIGURES

1.1 Tables

Operating in proximity of and on small bodies has proven particularly time consuming and challenging. Table S1 summarizes the autonomous capabilities used in past and planned in-space missions aimed at exploring small bodies.

Table S1. Highlights of autonomy advances across small-body missions (past and current)

	Demonstrated Autonomy Advance	Capability/ Technology	Key Gaps and Needed Capabilities
**1998 – 2001 Deep Space I**	Cruised autonomously for 3 of 36 months (<10%); 30-minute autonomous flyby	Planning/scheduling<br>Autonomous navigation (asteroid detection, orbit update, spacecraft low-thrust trajectory correction maneuvers)<br>System health management	**Key Gaps**<br>• Limited scope of autonomy use: capabilities have only been used for relatively short duration of the mission with pre- and sometimes post-monitoring from ground.<br>• Use of a priori maps: missions with proximity operations required extensive ground processing to generate maps that were used in subsequent autonomous maneuvers.<br>• Reliance on ground-based resource planning<br>**Needed Capabilities**<br>• End-to-end, long-duration autonomy<br>• Autonomy in light of faults and failures<br>• Autonomy in environments with large uncertainties and limited a priori knowledge of the environment<br>• Autonomy that can handle a wide range of conditions, adapt and learn from its operations
**2002 – 2011 Stardust**	30-minute autonomous flyby of one asteroid and two comets	Target-body detection (one body)<br>Orbit and attitude updates for tracking nucleus through flyby	
**2005 – 2010 Deep Impact**	Two-hour autonomous terminal guidance of comet impactor. Flyby tracking of two comets	Target-body detection (one body), orbit update, and spacecraft maneuvers	
**2005 Hayabusa**	Autonomous terminal descent of last 50 m toward a near-surface goal for sample collection	Laser ranging (at <100m) to adjust altitude and attitude	
**2019 Hayabusa**	Same as Hayabusa	Same as Hayabusa; bright surface object detection and centroiding; hybrid ground/onboard terminal descent control: ground controls boresight approach, while onboard controls lateral motion in final 50 m; on surface, open-loop control of surface hopping mobility	
**2020 OSIRIS-REx**	Terrain-relative navigation (TRN) for touch-and-go maneuver	Uses ground-generated shape-model, match natural features to model using TRN with ground planning; onboard adjustments to final maneuvers to initiate touch-and-go for sample collection	
**2022 DART**	Several hours of autonomous terminal guidance (similar to Deep Impact)	Identification of each body for target selection; thruster control to guidance impact; targeting the 170-m moon of a 780-m primary	

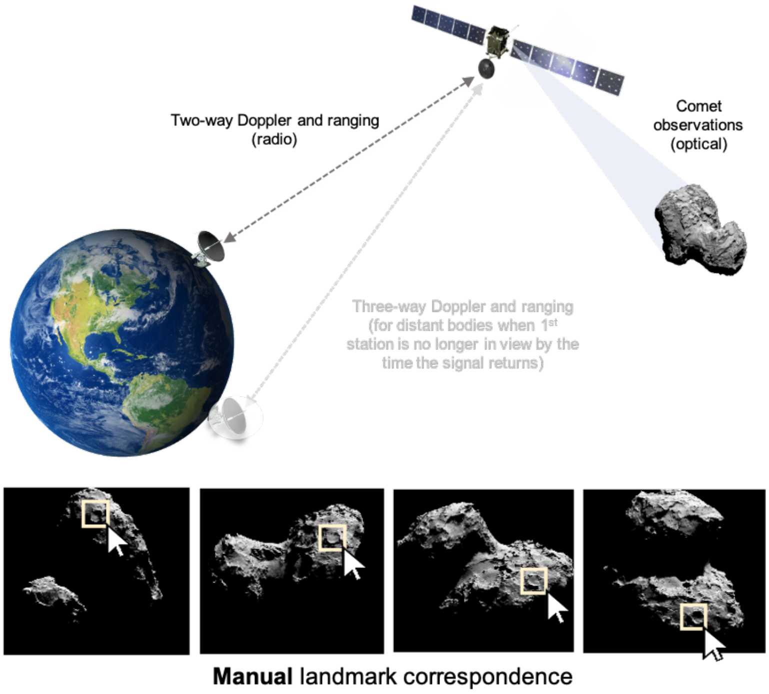

Figure S1. State of the practice in spacecraft navigation

1.2 Figures

Figure S1 shows a high-level overview of the process used in state of the practice for determining the orbit of the spacecraft relative to the body, which combines radiometric and optical data to plan approach and maneuvers for proximity operations, including landing on small bodies. The current and most widely used method to identify and track surface features is called Stereo Photoclinometry (SPC) (Gaskell et al., 2008), which is a ground-based semi-manual process that simultaneously refines the body landmarks and updates relative orbit.

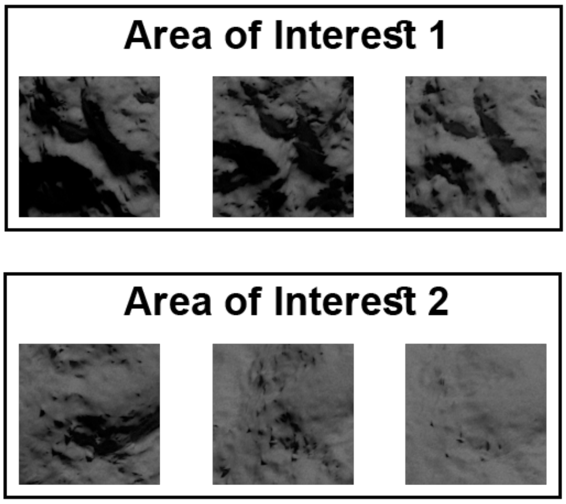

Figure S2. Image patches surrounding two surface features of a procedural-generated small body, as seen under different lighting and perspective conditions.

As the small body rotates, lighting causes dramatic changes in visual-appearance, which is accentuated by the absence of an atmosphere to diffuse light (see Figure S2),

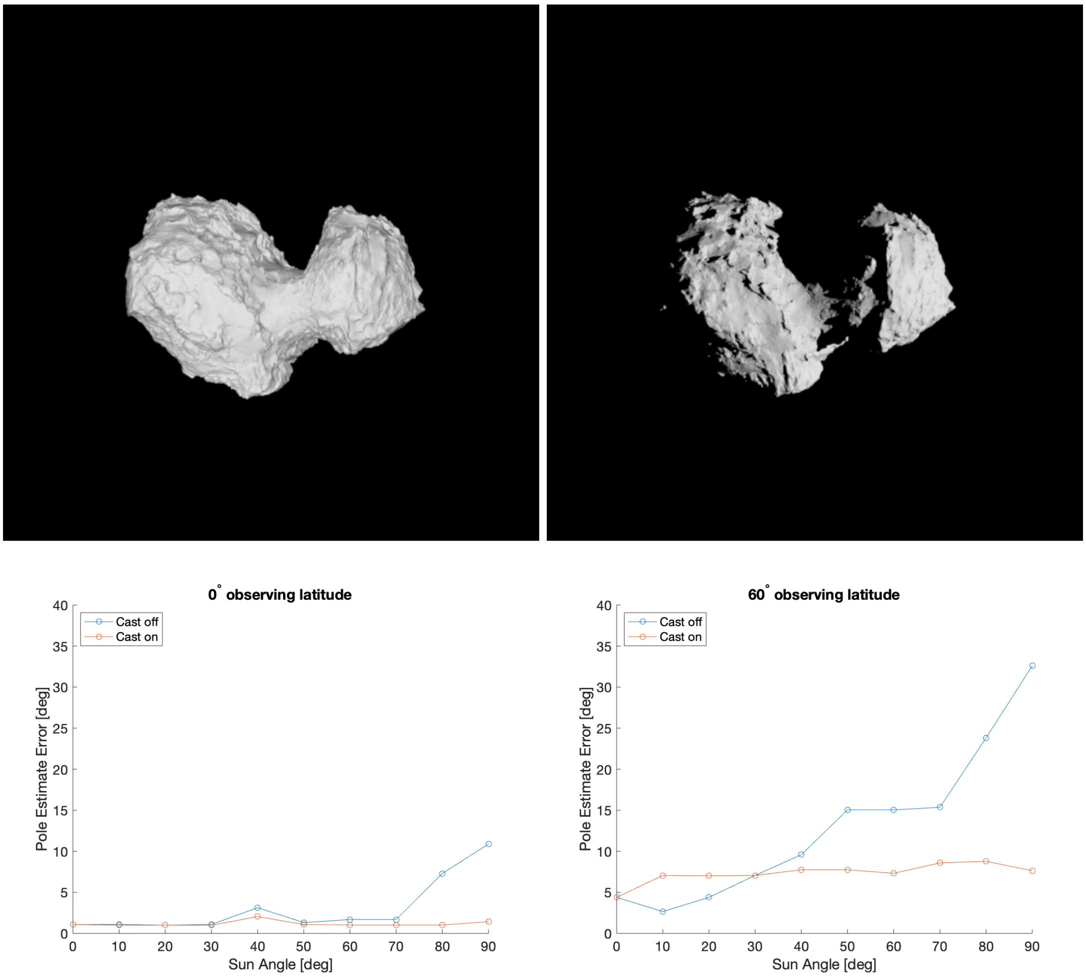

Figure S3. Top: Image rendering of Comet 67P with a $0°$ sun-phase angle (left) and a $60°$ sun-phase angle. **Bottom:** Pole error from the pole-from-silhouette (PfS) estimation algorithm at $0°$ sun-phase angle (left) and $60°$ sun-phase angle (right). The “Cast on” indicates the use of the shadow-casting variant of the pole-from-silhouette algorithm.

The performance of the pole-from-silhouette algorithm is affected by the sun-phase angle as well as the observing latitude. Figure S3(top) shows rendered images of Comet 67P/CG at two different sun-phase angles: $0°$ and $60°$ with their corresponding pole-estimate errors (Figure S3(bottom)). The errors are shown for two variants of the pole-from-silhouette algorithm: the ray-casting algorithm (Cast off in the figure) and a shadow-casting variant of the ray-casting algorithm (Cast on) that extends the performance of the algorithm and relaxes the assumption of a sun-phase angle of $< 20°$ for this algorithm. A more detailed description of these algorithms and an assessment of their performance can be found in (Bandyopadhyay et al., 2021).

Figure S4 shows the normalized mean Hausdorff distance for the coarse reconstruction of three bodies from a 1000-point dataset at different sun-phase angle using the Spherical Conformal Mapping approach with a body-symmetry assumption to recover information from shadowed regions. The results generally show a low percentage error in the coarse reconstruction (Jarvis et al., 2021).

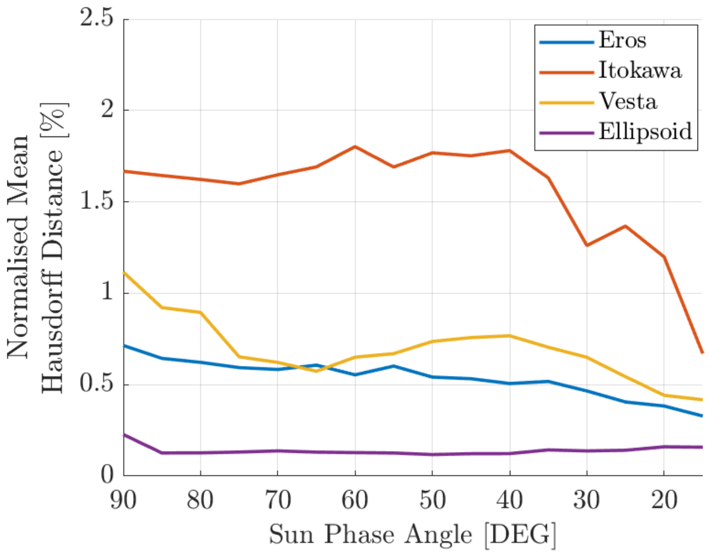

Figure S4. The performance of the coarse-shape reconstruction algorithm at different sun-phase angles.

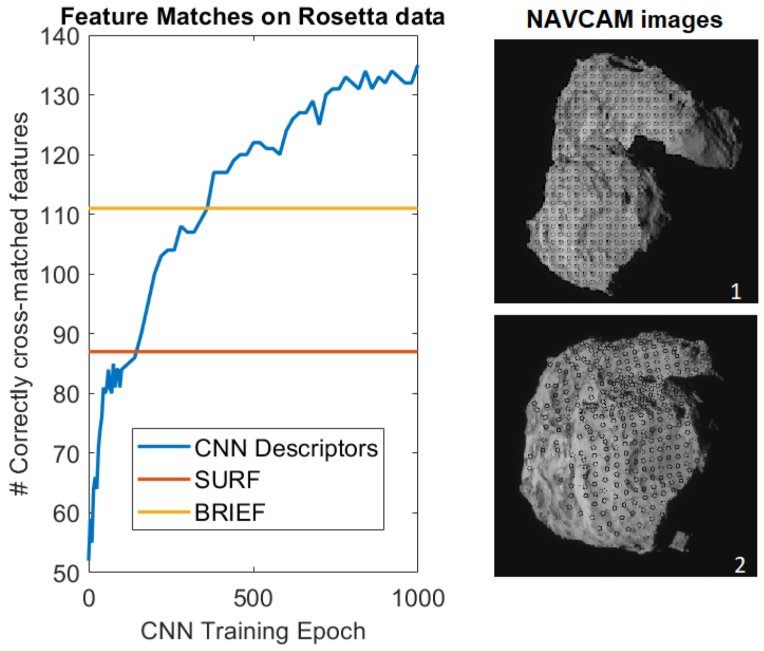

Figure S5. Correct matches on two NAVCAM images, from CNN, *SURF* and *BRIEF* descriptors (left). Features (grey dots) selected from image 1 and matched in image 2 from the CNN (right).

A Convolutional Neural-Network (CNN) image matcher is trained on synthetically generated bodies. Figure S2 shows an example of patches under different lighting and perspective channges, which are used to training the network. Figure S5 compares results of matching performance between classical feature-descriptor matching, SURF and BRIEF, and CNN-trained matching.

Our best estimate of the 6 Trajectory Correction Maneuvers (TCM) are shown in Table S2. All TCM are in EME2000 frame.

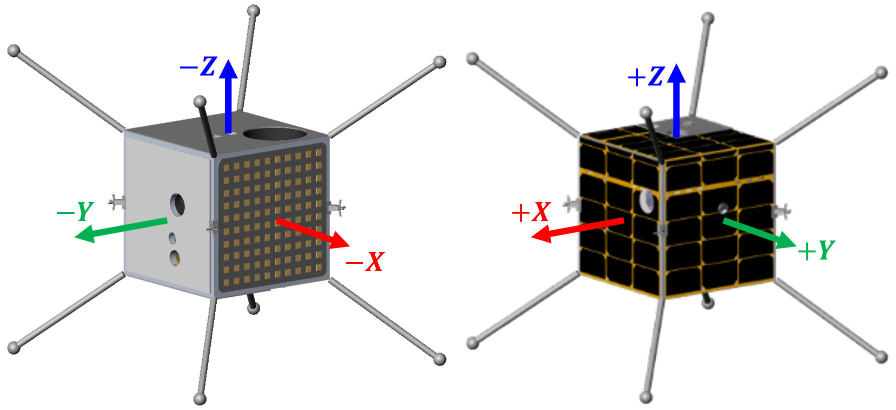

Face	Primary Function	Star Tracker	Sun Sensor	Camera	Insulated?
+ X	Solar array	Y	N	N	Y
+ Y	Solar Array	N	N	Y	Y
+ Z	Solar Array	Y	Y	N	Y
- X	Antenna	N	N	N	Y
- Y	Radiator	Y	Y	Y	N
- Z	Nadir imaging	N	N	Y	Y

Figure S6. External geometry of the notional 8U CubeSat architecture, consisting of solar arrays on three orthogonal faces, a phased array medium-gain antenna on one face, a radiator on another, and suite of imaging sensors on the final (nominally nadir-pointing) face.

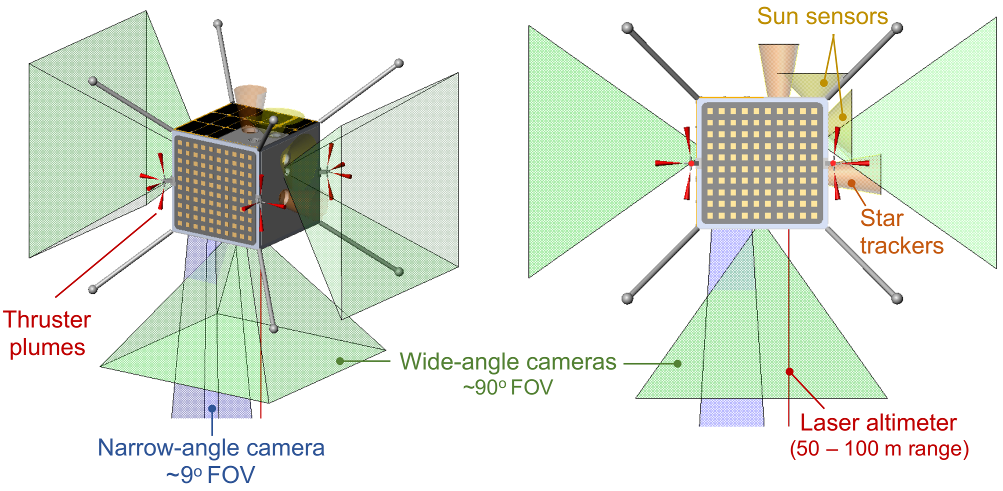

Figure S7. The cubic chassis is protected by eight corner-mounted “legs” for landing on any side. Optical frustums of spacecraft cameras, GNC sensors, as well as (red) thruster plumes are shown.

Table S2. Estimates of Trajectory Correction Maneuvers (TCM) during experiment

TCM	Date	Time	ΔV [km/sec]
#1	04-OCT-2022	00:00:00.0000 ET	[-0.002831429, 0.001469857, 0.000678015]
#2		12:00:00.0000 ET	[-0.005251283, 0.002736191, 0.002149652]
#3	05-OCT-2022	07:05:00.0000 ET	[-0.000295457, -0.000417792, -0.000853114]
#4		19:05:00.0000 ET	[0.000212417, 0.000273821, 0.000840847]
#5	06-OCT-2022	06:15:00.0000 ET	[0.000678541, -0.000376830, -0.000070059]
#6	07-OCT-2022	06:15:00.0000 ET	[-0.000688199, 0.000318379, 0.000072940]

REFERENCES

Bandyopadhyay, S., Villa, J., Osmundson, A., Hockman, B., Morrell, B., et al. (2021). Light-robust pole-from-silhouette algorithm and visual-hull estimation for autonomous optical navigation to an unknown body. In *AAS Guidance, Navigation and Control Conference* (Breckenridge, Colorado), 1

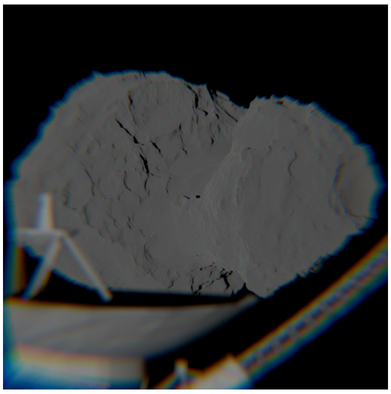

Figure S8. Synthetic image showcasing exaggerated camera artifacts, with spacecraft components in the foreground and comet 67P in the background. The Lommel-Seeliger model is used to render the comet in this frame.

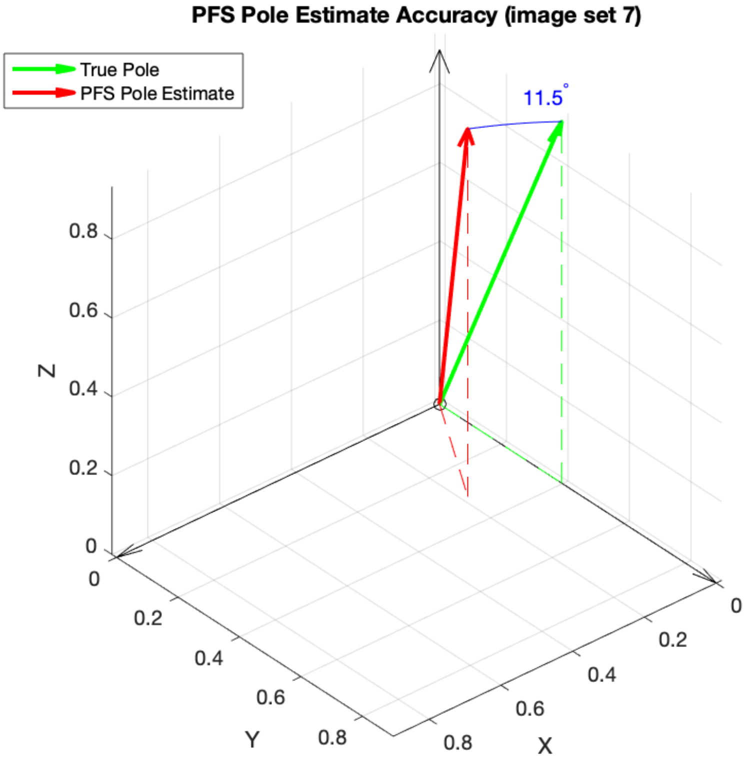

Figure S9. Error in pole estimate from running pole-from-silhouette algorithm on image set 7

Gaskell, R., Barnouin-Jha, O., Scheeres, D. J., Konopliv, A., Mukai, T., Abe, S., et al. (2008). Characterizing and navigating small bodies with imaging data. *Meteoritics & Planetary Science* 43, 1049–1061

Jarvis, B., Choi, G. P. T., Hockman, B., Morrell, B., Bandopadhyay, S., Lubey, D., et al. (2021). 3D shape reconstruction of small bodies from sparse features. *IEEE Robotics and Automation Letters* , 7089–7096doi:10.1109/LRA.2021.3097273

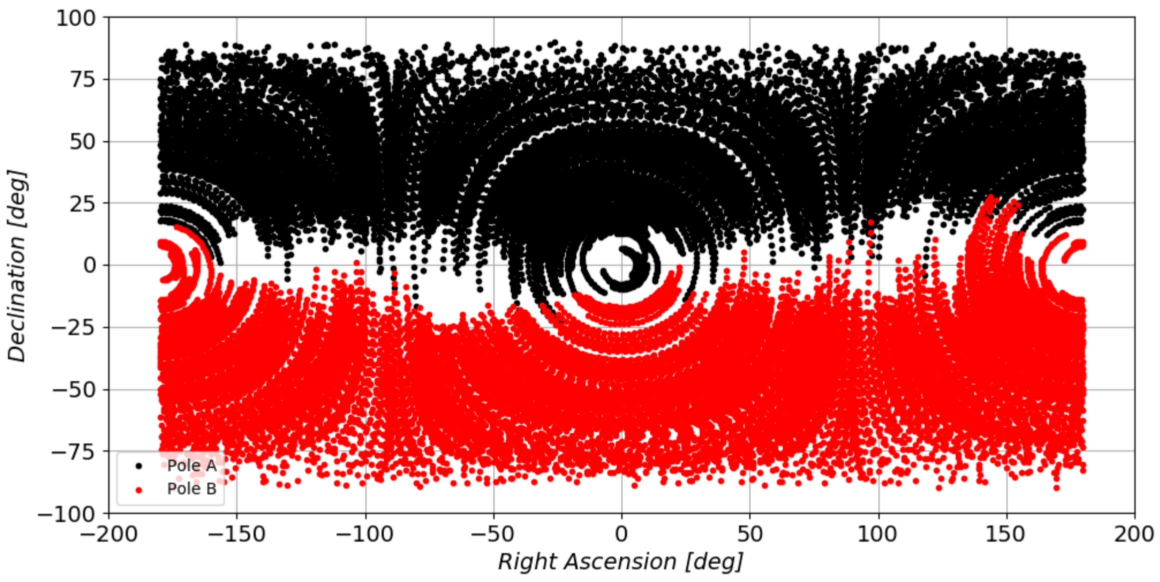

Figure S10. Landmark angles relative to the body's coordinate frame for two pole hypotheses (black and red) from image sets 9–14. The body's coordinate frame has $+z$-axis pointing from body's center of mass toward the spacecraft. Landmarks with positive-declination angles (black) are on the observable side of the body (between the body center and the camera), while landmarks with negative-declination angles (red) are on the opposite side, which would get obstructed from view by the body ($-z$-axis of the body-camera line). Positive declination angles disambiguate different pole hypotheses that result from the Pos "ballerina effect."

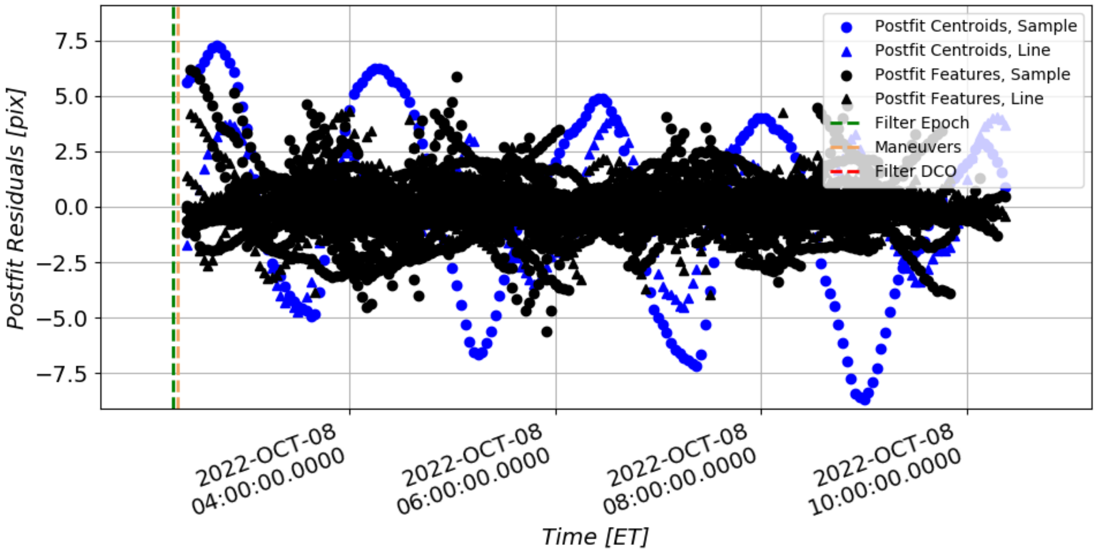

Figure S11. Example of postfit residuals for centroid and feature tracking.
